# A Bayesian perspective on observers’ inference of group norms

**DOI:** 10.1038/s41539-026-00405-x

**Published:** 2026-03-02

**Authors:** Jipeng Duan, Xiuyan Guo, Li Zheng, Jun Yin

**Affiliations:** 1 Department of Psychology, Ningbo University, Ningbo, China; 2 Center of Group Behavior and Social Psychological Service, Ningbo University, Ningbo, China; 3 Fudan Institute on Ageing, Fudan university, Shanghai, China; 4 Ministry of education (MOE) Laboratory for National Development and Intelligent Governance, Fudan university, Shanghai, China

**Keywords:** Psychology, Psychology

## Abstract

Inferring group norms is crucial for adapting behaviors in novel situations, but its underlying basis and computational account remain unclear. This study manipulated the prevalence of norm-consistent behaviors (i.e., straight-line movements) to examine whether and how norm inference is influenced by observed group behavior, exploring its consistency with Bayesian updating, robustness, and independence. The results revealed no significant difference in prior probabilities regarding the existence of group norms across conditions, but posterior probabilities increased with the prevalence of norm-consistent behaviors. Furthermore, the Bayesian inference model outputs positively predicted participants’ judgments, indicating that norm inference aligned with Bayesian updating. Even in the presence of deviant behaviors, norm inference remained consistent with Bayesian principles, demonstrating its robustness. Finally, the study revealed that individuals could infer group norms from observed behaviors, independent of desire inferences. These findings enhance our understanding of how individuals navigate group norms in novel situations.

## Introduction

In daily life, individuals usually engage in behaviors consistent with those of other members of the group, and tend to comply with group norms^1,2^. Complying with group norms not only contributes to individuals’ adaptation to novel or changeable environments^3,4^ but also facilitates or maintains prosocial behaviors, including cooperation and mutual support among group members^5^, and even the establishment of organizational responses to global environmental crises—for example, encouraging curbside recycling programs through peer-influenced norms^6^. On the basis of these findings, compliance with group norms is clearly essential for both individuals and communities. However, complying with group norms is founded on judging and identifying the norms (i.e., the inference of group norms), which forms the foundation for individuals to assimilate into social situations and exhibit adaptive behaviors^3,7^. What, then, is the basis for the norm inferences? How are the norm inferences carried out? What characteristics does it exhibit? These questions require further exploration.

A few existing studies have provided insights into the inference of group norms^8,9^. For instance, through a risky decision-making task, Reiter et al.^8^ reported that members of social groups initially displayed a risk-averse tendency and made more conservative choices. However, once participants observed others in the group frequently engaging in risky behaviors, their risk preferences subsequently changed, leading them to exhibit more risk-seeking behavior. The results indicated that when other group members consistently engaged in a particular behavior, participants were likely to perceive this behavior as a group norm and adjust their own actions accordingly, suggesting that the consistent behavior exhibited by others may be a key basis underpinning an individual’s inference of group norms. This is because once group norms, which act as shared constraints on group members’ behaviors, exist, almost all members of the group will exhibit consistent norm-following behaviors^2,10,11^.

Given this, how do individuals infer group norms on the basis of the observed consistent behaviors of other members? Existing simulation-based studies indicate that the inference of group norms can be conceptualized as the updating of norm-related beliefs consistent with Bayesian principles^12^. Bayesian principles are particularly suited for explaining norm inference for two main reasons. First, it assumes that perceivers act as “lay Bayesians”, integrating new cues with their prior knowledge to update beliefs^13^, which mirrors the hypothesis that norm inference is consistent with Bayesian belief updating based on observed group behavior^12^. Second, it provides a principled framework for inferring latent states of others or the environment from observable outcomes. Social norms are abstract, hidden constraints on group behavior^14^, and Bayesian principles provide a computational account of how people make judgments about such latent states by observing others’ actions. Accordingly, following the methodological approach of prior research^15^, the present study aims to evaluate the performance of human individuals in norm inference tasks and investigate whether actual norm inferences are consistent with Bayesian updating.

Owing to the diversity of group behavior, group members may exhibit deviant behaviors that are inconsistent with group norms. To examine whether group norm inferences are consistent with Bayesian updating in the presence of negative evidence, deviant behaviors were introduced. Specifically, in addition to behaviors consistent with group norms, several individuals may exhibit deviant behaviors inconsistent with norms^16,17^. With respect to how deviant behaviors influence group norm inferences within the framework of Bayesian updating, two possible scenarios exist. On the one hand, the presence of deviant behaviors may suggest that group members disagree with the collective recognition of the group (such as group function or goals), indicating the emergence of group disidentification, which impairs perceptions of group entitativity and undermines individuals’ belief about the group’s existence^18^. Ultimately, the presence of deviant behaviors may hinder the Bayesian inference of norms grounded in the existence of the group. On the other hand, individuals infer group norms by observing the behaviors of others in the group, primarily to facilitate their own adaptive behaviors in the current situation^19^. This need leads individuals to focus on specific behavioral performances without overthinking the meanings (e.g., attitudes of the actor) underlying them^20,21^. Therefore, although deviant behaviors may affect posterior probabilities and the updating degree of norm-related beliefs, these measures may generally still increase with the prevalence of norm-consistent behaviors, allowing norm inference to remain consistent with Bayesian updating. This study introduced deviant behaviors to examine the robustness of norm inference consistent with Bayesian principles.

Notably, when group members engage in specific behaviors, it could reflect their personal desires^22,23^, in addition to complying with group norms. By observing others’ behaviors in groups, individuals can infer not only group norms but also the desires underlying those behaviors^24^. For example, after observing that people dispose of trash by placing it in bins, it could be inferred that this behavior is due to both complying with group norms and/or a personal desire to keep the environment clean. To investigate the relationship between these two types of inferences, measurements of group members’ desires were included in the present study. With respect to the relationship between these two types of inferences, three possible scenarios exist. First, the inference of group norms based on observed behaviors may be underpinned by the inference of desires. Researchers have suggested that once internalized, group norms can influence individuals’ behavioral performance by altering their desires^25^, implying that inferring group norms from observed behaviors may rely on the inference of desires. Second, the inference of group norms may be independent of the inference of desires. Specifically, in addition to recognizing individual mental states (e.g., desires), people can identify the collective recognition of group members, a capacity known as shared intentionality^26^. This ability allows individuals to regard behaviors exhibited by multiple members as indicators of group identification and perceive these behaviors as group norms, which they accept upon joining the group^27^, sometimes even overriding personal desires^28–30^. Given these facts, it is possible that the ability of shared intentionality enables individuals to infer group norms directly without speculating about members’ mental states (such as desires). Finally, both inferences may be simultaneously available, meaning that the inferences of group norms can occur both depending on the inferences of desires and independently of them. Thus, considering the potential relationship between these two types of inferences, the present study aims to investigate whether the inference of group norms is independent of the inference of desires, further confirming the characteristics of the inference of group norms.

To address the above issues, in reference to previous studies^31,32^, the current study investigated the inference of group norms by asking participants to observe computer-animated events in which five cartoon characters formed groups and displayed specific behaviors. This method allows for strict control of potential confounding factors and has been widely used in social cognition^33,34^. Notably, while the experimental manipulation provides participants with descriptive evidence (i.e., the number or proportion of group members exhibiting certain behaviors), the primary objective of this study is to investigate how individuals—in the absence of explicit normative instructions—utilize these observed behavioral regularities to infer the underlying shared normative constraints. This process is more closely aligned with the identification of injunctive norms, which characterize the shared expectations of what one ought to do in a novel group situation^35^.

In summary, the current study consists of three experiments. Experiment 1 is designed to investigate whether individuals can infer group norms on the basis of the observed consistent behaviors of group members and whether the inference is consistent with Bayesian updating. Moreover, Experiment 2 aims to examine the robustness of group norm inferences by introducing deviant behaviors that are inconsistent with the group norms. Finally, on the basis of the possible relationship between the inference of group norms and the inference of desires, by constructing different Bayesian network models and comparing the predictive effects of these models on participants’ norm-related judgments, Experiment 3 aims to explore whether the inference of group norms is independent of the inference of desires, thereby further confirming the characteristics of group norm inferences.

## Results

### Experiment 1—prior probabilities, posterior probabilities and updating degrees of norm-related belief

Experiment 1 compared the prior probabilities, the posterior probabilities, and the updating degrees of belief related to the prior and posterior probabilities (i.e., *D*_*KL*_) under different conditions. If the prior probabilities show no differences, whereas the posterior probabilities and the updating degrees of belief are influenced by the number of norm-consistent behaviors, it would provide evidence that participants can make the inference of norms on the basis of the observed behaviors of multiple group members.

**Fig. 1:**
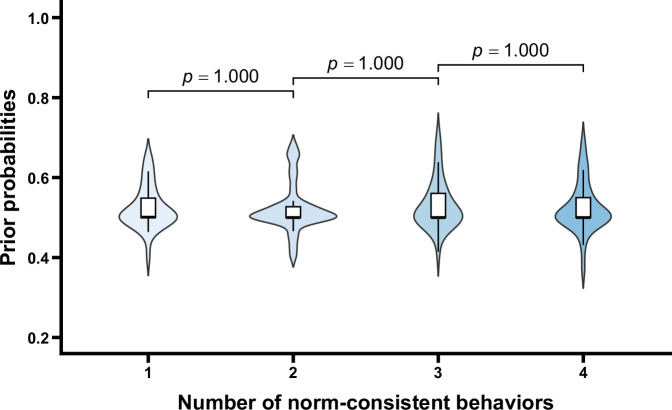
Prior probabilities as a function of the number of norm-consistent behaviors. Violin plots and box plots are presented for each condition. Exact *p* values shown in the figure reflect comparisons between adjacent conditions.

The prior probabilities under different conditions are shown in Fig. 1. One-way repeated measures ANOVA indicated that the main effect of the number of norm-consistent behaviors was not significant, *F* (2.06, 63.85) = 0.73, *p* = 0.491. Additionally, one-sample *t* tests comparing the prior probabilities under each condition against 0.5—representing a state of uncertainty regarding the existence of potential norms—revealed no significant differences, *t*s < 2.91, *p*s > 0.079. These results suggest that before different numbers of group members exhibiting normative behaviors were observed, the prior probabilities judged by the participants did not significantly differ across conditions and were indistinguishable from 0.5. Therefore, it is evident that before observing the behaviors of group members, participants were uncertain about which of the potential norms was applicable. Importantly, when controlling for gender and age, the main effect of the number of norm-consistent behaviors remained unchanged (*p* = 0.491). Neither gender, age, nor their interactions with the number of norm-consistent behaviors reached significance (*p*s > 0.137), indicating that the observed pattern was not influenced by participants’ gender or age (see the Supplementary Materials for details).

**Fig. 2:**
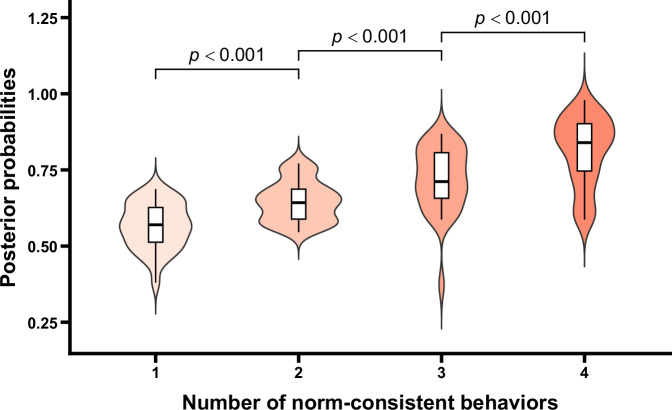
Posterior probabilities as a function of the number of norm-consistent behaviors. Violin plots and box plots are presented for each condition. Exact *p* values shown in the figure reflect comparisons between adjacent conditions.

The posterior probabilities under different conditions are shown in Fig. 2. One-way repeated measures ANOVA revealed that the main effect of the number of norm-consistent behaviors was significant, *F* (1.48, 45.96) = 67.16, *p* < 0.001, *η*_p_^2^ = 0.68. Further post-hoc comparisons indicated that there were significant differences in the posterior probabilities between the adjacent conditions of number of norm-consistent behaviors, *t*s > 5.31, *p*s < 0.001, Cohen’s *d*s > 0.72. Additionally, one-sample *t* tests comparing the posterior probabilities under each condition to 0.5 revealed that, in all conditions, the posterior probabilities were significantly greater than 0.5, *t*s > 4.92, *p*s < 0.001, Cohen’s *d*s > 0.87. These results indicated that participants’ judgmental posterior probabilities increased with the number of norm-consistent behaviors, suggesting that the observed norm-consistent behaviors of group members serve as the basis for participants’ inference of group norms. Importantly, after controlling for gender and age, the main effect of the number of norm-consistent behaviors remained significant (*p* < 0.001). Neither gender, age, nor their interactions with the number of norm-consistent behaviors were significant (*p*s > 0.491), indicating that these demographic factors did not influence the observed pattern of results (see the Supplementary Materials for details).

**Fig. 3:**
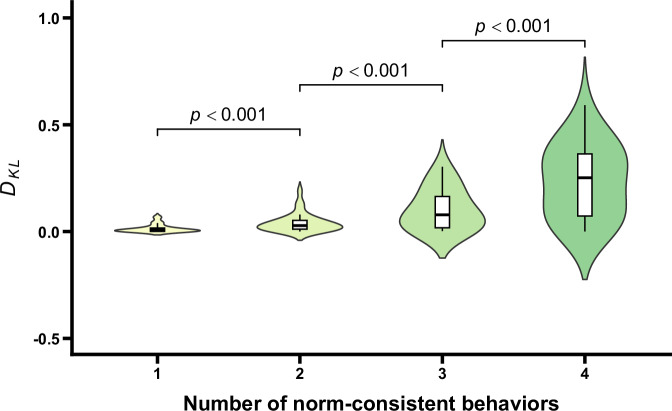
D_KL_ as a function of the number of norm-consistent behaviors. Violin plots and box plots are presented for each condition. Exact *p* values shown in the figure reflect comparisons between adjacent conditions.

The updating degrees of norm-related belief (i.e., *D*_*KL*_) under different conditions are shown in Fig. 3. The ANOVA results revealed that the main effect of the number of norm-consistent behaviors was significant, *F* (1.21, 37.39) = 49.76, *p* < 0.001, *η*_p_^2^ = 0.62. Further post-hoc comparisons revealed significant differences in updating degrees of norm-related belief between the adjacent conditions of the number of norm-consistent behaviors, *t*s > 4.82, *p*s < 0.001, Cohen’s *d*s > 0.24. Additionally, given that a *D*_*KL*_ value of 0 indicates that there is no difference between the probability distributions, implying no updating in the norm-related belief, one-sample *t* tests comparing the *D*_*KL*_ under each condition to 0 were conducted. The results revealed that the *D*_*KL*_ under all conditions was significantly different from 0, *t*s > 4.20, *p*s < 0.003, Cohen’s *d*s > 0.74. All these results indicated that updating degrees of norm-related belief increased with the number of norm-consistent behaviors. The results related to the prior probabilities, the posterior probabilities, and the updating degrees of norm-related belief consistently showed that participants inferred group norms on the basis of the observed behaviors of group members. Importantly, after controlling for gender and age, the main effect of the number of norm-consistent behaviors remained significant (*p* < 0.001). Neither gender, age, nor their interactions with the number of norm-consistent behaviors were significant (*p*s > 0.409), indicating that demographic factors did not influence the observed effects (see the Supplementary Materials for details).

### Experiment 1—prediction of model-derived posterior probabilities on participants’ judgmental posterior probabilities

Furthermore, Experiment 1 explored the relationship between the model-derived posterior probabilities and the participants’ judgmental posterior probabilities. If the former can positively predict the latter, it would indicate that the inference of group norms is consistent with Bayesian updating.

**Fig. 4:**
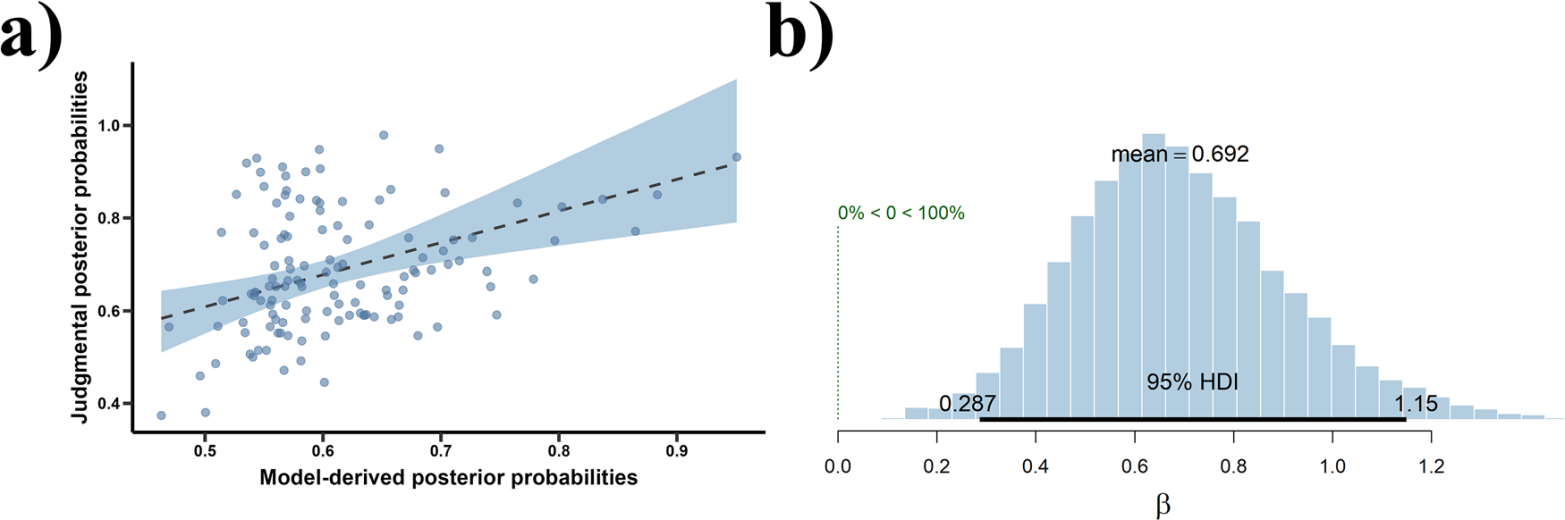
Results of the Bayesian hierarchical linear regression model for model-derived posterior probabilities predicting judgmental posterior probabilities. **a** The positive prediction effect of model-derived posterior probabilities on participants’ judgmental posterior probabilities. The blue region indicates the 95% confidence interval corresponding to *β*. **b** Posterior distribution of *β* drawn from the Bayesian hierarchical linear regression model. In this figure, the 95% HDI represents 95% highest density interval with the same meaning as the 95% CrI. The legend “0% < 0 < 100%” visually depicts the posterior probability mass for the slope parameter *β* that falls above or below zero, corresponding to *Pr* (*β* > 0) in the data.

As shown in Fig. 4a, b, the results of the Bayesian hierarchical linear regression model with a random effect for each participant indicate that the model-derived posterior probabilities positively predict the participants’ judgmental posterior probabilities, *β* = 0.69, 95% CrI = [0.29, 1.15], *Pr* (*β* > 0) = 100.00%, *δ*_t_ = 8.31. Moreover, because Rhat is equal to 1, the Bayesian hierarchical linear regression model has converged. These results suggest that the inference of group norms is consistent with Bayesian updating.

Moreover, although a Bayesian hierarchical linear regression model, with a random effect for each participant, revealed that the Frequency tracking model could also positively predict participants’ actual judgments, *β* = 0.48, 95% CrI = [0.38, 0.58], *Pr* (*β* > 0) = 100.00%, *δ*_t_ = 1.98, closer examination revealed that its predictive output was coarse, producing only a limited set of fixed estimates that clustered the data (see Supplementary Fig. 1b). This meant that it treated distinct responses as indistinguishable, missing the subtle variability in participants’ judgments. In contrast, the Bayesian model generated a continuous range of estimates, which better captured the nuanced variability.

To further test model performance, the experimental condition was added as a random variable to the hierarchical regression model. Under this more rigorous test, the Frequency tracking model could no longer positively predict participants’ responses, *β* = 0.40, 95% CrI = [−3.31, 4.80], *Pr* (*β* > 0) = 78.20%, *δ*_t_ = 2.94, whereas the Bayesian model continued to do so, *β* = 0.49, 95% CrI = [−0.04, 0.99], *Pr* (*β* > 0) = 96.80%, *δ*_t_ = 1.64. These results revealed that the Bayesian inference model provided a nuanced predictive performance for participants’ responses, offering stronger evidence for our hypothesis (see the Supplementary Materials for model construction and comparison details).

### Experiment 2—prior probabilities, posterior probabilities and updating degrees of norm-related belief

Similar to Experiment 1, if the prior probabilities show no differences across conditions, while the posterior probabilities and the updating degrees of norm-related belief increase with the proportion of norm-consistent behaviors overall, it would indicate that participants still infer group norms on the basis of the observed behaviors of group members, even in the presence of deviant behaviors as negative evidence.

**Fig. 5:**
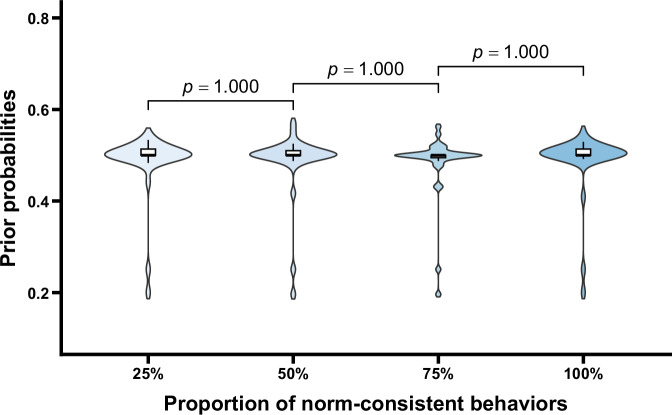
Prior probabilities as a function of the proportion of norm-consistent behaviors. Violin plots and box plots are presented for each condition. Exact *p* values shown in the figure reflect comparisons between adjacent conditions.

The prior probabilities under different conditions are shown in Fig. 5. One-way repeated measures ANOVA revealed that the main effect of the proportion of norm-consistent behaviors was not significant, *F* (1.55, 47.90) = 0.37, *p* = 0.639. Additionally, one-sample *t* tests were conducted to compare the prior probabilities under each condition to 0.5, the results showed that there were no significant differences, *t*s < 1.38, *p*s = 1.000 (corrected *p* values). These results indicated that before observing the behaviors of group members, participants’ judgmental prior probabilities did not show significant differences across conditions and were indistinguishable from 0.5, suggesting that participants were uncertain about which of the potential norms applied in the situations. Importantly, after controlling for gender and age, the main effect remained unchanged (*p* = 0.913). Neither gender, age, nor their interactions with the proportion of norm-consistent behaviors were significant (*p*s > 0.084), indicating that the pattern of results was not influenced by participants’ gender or age (see the Supplementary Materials for details).

**Fig. 6:**
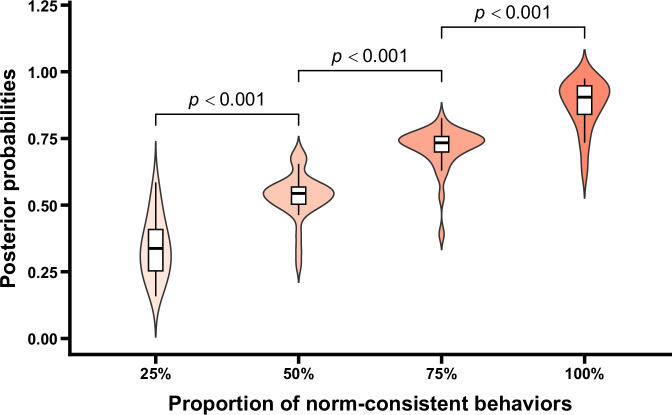
Posterior probabilities as a function of the proportion of norm-consistent behaviors. Violin plots and box plots are presented for each condition. Exact *p* values shown in the figure reflect comparisons between adjacent conditions.

The posterior probabilities under different conditions are shown in Fig. 6. One-way repeated measures ANOVA revealed a significant main effect for the proportion of norm-consistent behaviors, *F* (1.40, 43.39) = 287.92, *p* < 0.001, *η*_p_^2^ = 0.90. Further post-hoc comparisons indicated significant differences in the posterior probabilities between adjacent conditions, *t*s > 12.12, *p*s < 0.001, Cohen’s *d*s > 1.79. Additionally, one-sample *t* tests were conducted for the posterior probabilities in each condition against 0.5. The results showed that when only 25% of the sample agents exhibited the norm-consistent behaviors, the posterior probabilities judged by participants were significantly lower than 0.5, *t* (31) = −8.06, *p* < 0.001, Cohen’s *d* = −1.42. When 50% of the sample agents exhibited norm-consistent behaviors, there was no significant difference between the posterior probabilities and 0.5, *t* (31) = 2.25, *p* = 0.379. In contrast, when 75% or 100% of the sample agents performed the norm-consistent behaviors, the posterior probabilities were significantly higher than 0.5, *t*s > 14.75, *p*s < 0.001, Cohen’s *d*s > 2.61. These results collectively indicated that although posterior probabilities were influenced by the deviant behaviors (e.g., posterior probabilities at 25% condition were lower than 0.5), they still increased with the proportion of norm-consistent behaviors. When the majority of group members exhibited norm-consistent behaviors, participants believed that the probabilities of existence of norms were higher than 0.5. Thus, it can be inferred that participants still infer group norms based on the observed behaviors, even in the presence of the deviant behaviors as negative evidence. Importantly, controlling for gender and age did not alter the main effect of the proportion of norm-consistent behaviors (*p* < 0.001). Neither gender, age, nor their interactions with the proportion of norm-consistent behaviors were significant (*p*s > 0.170), indicating that the observed pattern was not affected by participants’ demographic characteristics (see the Supplementary Materials for details).

**Fig. 7:**
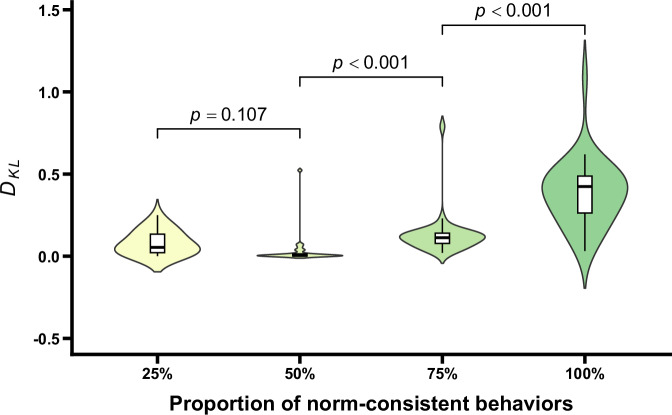
D_KL_ as a function of the proportion of norm-consistent behaviors. Violin plots and box plots are presented for each condition. Exact *p* values shown in the figure reflect comparisons between adjacent conditions.

The updating degrees of norm-related belief (i.e., *D*_*KL*_) under different conditions are shown in Fig. 7. One-way repeated measures ANOVA revealed a significant main effect for the proportion of norm-consistent behaviors, *F* (1.91, 59.12) = 88.64, *p* < 0.001, *η*_p_^2^ = 0.74. Further post-hoc comparisons indicated that the *D*_*KL*_ at 50% norm-consistent behaviors did not significantly differ from that at 25%, *t* (31) = -2.50, *p* = 0.107. In contrast, the *D*_*KL*_ for 75% norm-consistent behaviors was significantly greater than that for 50%, *t* (31) = 9.96, *p* < 0.001, Cohen’s *d* = 0.74, and the *D*_*KL*_ for 100% norm-consistent behaviors was also significantly greater than that for 75%, *t* (31) = 11.48, *p* < 0.001, Cohen’s *d* = 1.91. Moreover, one-sample *t* tests comparing *D*_*KL*_ under each condition to 0 (indicating no updating of norm-related belief) showed that only the *D*_*KL*_ for 50% norm-consistent behaviors did not significantly differ from 0, *t* (31) = 1.81, *p* = 0.951. In contrast, *D*_*KL*_ for all other conditions were significantly greater than 0, *t*s > 5.58, *p*s < 0.001, Cohen’s *d*s > 0.99. These results indicated that after observing the minority (25%) of group members exhibiting the norm-consistent behaviors, participants updated their norm-related belief; however, judged probabilities of existence of group norms decreased. Conversely, when the majority (75% or 100%) of group members exhibited norm-consistent behaviors, participants also updated their norm-related belief, resulting in a significant increase in the judged probabilities of the existence of group norms. Collectively, all results, including the prior probabilities and posterior probabilities, suggest that the presence of deviant behaviors does not undermine group norm inferences, individuals still infer group norms based on the observed behaviors. Importantly, controlling for gender and age did not affect the main effect of the proportion of norm-consistent behaviors (*p* < 0.001). Neither gender nor age, nor their interactions with the proportion of norm-consistent behaviors were significant (*p*s > 0.636), indicating that the pattern of results was not influenced by participants’ gender or age (see the Supplementary Materials for details).

### Experiment 2—prediction of model-derived posterior probabilities on participants’ judgmental posterior probabilities

Furthermore, if the model-derived posterior probabilities can positively predict the participants’ judgmental posterior probabilities, it would confirm that the inference of group norms is still consistent with Bayesian updating despite the presence of deviant behaviors.

**Fig. 8:**
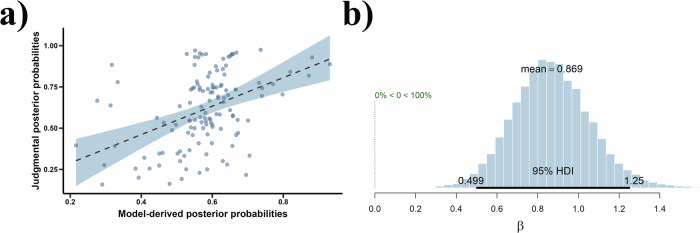
Results of the Bayesian hierarchical linear regression model for model-derived posterior probabilities predicting judgmental posterior probabilities. **a** The positive prediction effect of model-derived posterior probabilities on participants’ judgmental posterior probabilities. The blue region indicates the 95% confidence interval corresponding to *β*. **b** Posterior distribution of *β* drawn from the Bayesian hierarchical linear regression model. In this figure, the 95% HDI represents 95% highest density interval with the same meaning as the 95% CrI. The legend “0% < 0 < 100%” visually depicts the posterior probability mass for the slope parameter *β* that falls above or below zero, corresponding to *Pr* (*β* > 0) in the data.

As shown in Fig. 8a, b, the results of Bayesian hierarchical linear regression model indicated that model-derived posterior probabilities positively predicted participants’ judgmental posterior probabilities, *β* = 0.87, 95% CrI = [0.50, 1.25], *Pr* (*β* > 0) = 100.00%, *δ*_t_ = 16.50. Moreover, since Rhat equaled 1, it is evident that the Bayesian hierarchical linear regression model converged. Therefore, even in the presence of deviant behaviors as negative evidence, participants’ inference of group norms was still consistent with Bayesian updating.

Meanwhile, although a Bayesian hierarchical linear regression model, with a random effect for each participant, showed that Frequency tracking model also could positively predict participants’ actual judgments, *β* = 0.48, 95% CrI = [0.38, 0.58], *Pr* (*β* > 0) = 100.00%, *δ*_t_ = 1.98, a closer inspection revealed that its predictive output was coarse, producing only a limited set of clustered estimates. When a more rigorous test was applied by adding the experimental condition as a random variable to the hierarchical regression model, the Frequency tracking model was no longer able to positively predict participants’ responses, *β* = 0.87, 95% CrI = [−2.36, 3.91], *Pr* (*β* > 0) = 86.50%, *δ*_t_ = 3.08. In contrast, the Bayesian inference model continued to do so, *β* = 0.31, 95% CrI = [−0.02, 0.63], *Pr* (*β* > 0) = 96.90%, *δ*_t_ = 0.86. The results revealed that the Bayesian inference model provided a nuanced predictive performance for participants’ responses.

### Experiment 3—prior probabilities, posterior probabilities and updating degrees of norm-related belief

Consistent with the previous experiments, if the prior probabilities do not differ across conditions, while the posterior probabilities and the updating degrees of norm-related belief are influenced by the proportion of norm-consistent behaviors, it would indicate that participants are capable of inferring group norms based on the observed behaviors.

**Fig. 9:**
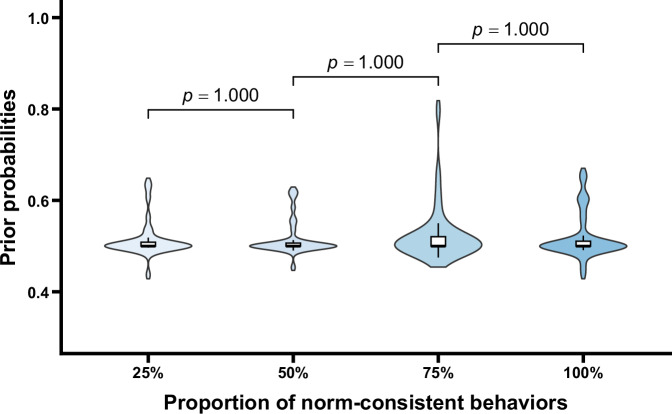
Prior probabilities as a function of the proportion of norm-consistent behaviors. Violin plots and box plots are presented for each condition. Exact *p* values shown in the figure reflect comparisons between adjacent conditions.

The prior probabilities under different conditions are shown in Fig. 9. One-way repeated measures ANOVA indicated no significant main effect of the proportion of norm-consistent behaviors, *F* (2.28, 70.74) = 0.65, *p* = 0.544. Additionally, one-sample *t* tests comparing the prior probabilities in each condition to 0.5 revealed no significant differences, *t*s < 2.41, *p*s > 0.268. These findings suggest that before observing group members’ behaviors, the prior probabilities judged by the participants did not differ significantly across conditions and were indistinguishable from 0.5, reflecting uncertainty about which norm applied. Importantly, after controlling for gender and age, the main effect of the proportion of norm-consistent behaviors remained unchanged (*p* = 0.832). Neither gender, age, nor their interactions with the proportion of norm-consistent behaviors were significant (*p*s > 0.639), indicating that the observed pattern of results was consistent across participants’ demographic characteristics (see the Supplementary Materials for details).

To highlight the distinction between norm-related and desire-related measures, the descriptive statistics for posterior probabilities of both are presented in Table 1.

**Table 1:** Descriptive statistics for norm- and desire-related posterior probabilities (*M* ± *SD*)

The proportion of norm-consistent behaviors	Norm	Desire
25%	0.32 ± 0.13	0.55 ± 0.20
50%	0.57 ± 0.09	0.62 ± 0.10
75%	0.75 ± 0.08	0.68 ± 0.11
100%	0.85 ± 0.09	0.74 ± 0.18

**Fig. 10:**
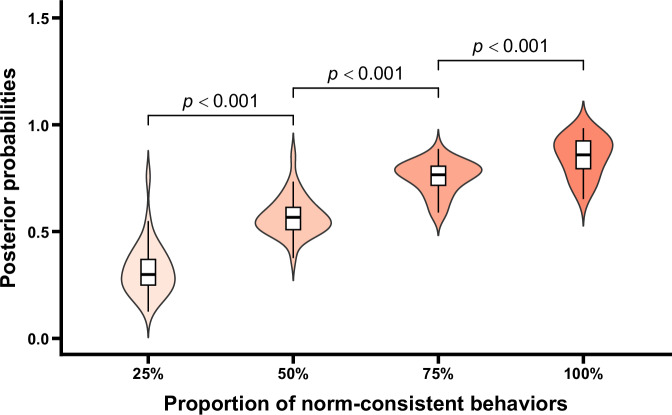
Posterior probabilities as a function of the proportion of norm-consistent behaviors. Violin plots and box plots are presented for each condition. Exact *p* values shown in the figure reflect comparisons between adjacent conditions.

The norm-related posterior probabilities under different conditions are shown in Fig. 10. One-way repeated measures ANOVA revealed a significant main effect of the proportion of norm-consistent behaviors, *F* (1.64, 50.94) = 252.53, *p* < 0.001, *η*_p_^2^ = 0.89. Further post hoc comparisons indicated significant differences in posterior probabilities between adjacent conditions, *t*s > 9.19, *p*s < 0.001, Cohen’s *d*s > 0.85. One-sample *t* tests comparing the posterior probabilities in each condition to 0.5 showed that when only 25% of the sample agents exhibited norm-consistent behaviors, the judged probability of the existence of group norms was significantly lower than 0.5, *t* (31) = −8.11, *p* < 0.001, Cohen’s *d* = −1.43. In contrast, when 50%, 75% or 100% of the sample agents exhibited the norm-consistent behaviors, the probabilities of existence of group norms judged by participants were significantly higher than 0.5, *t*s > 4.46, *p*s < 0.002, Cohen’s *d*s > 0.79. These results showed that while posterior probabilities were influenced by the deviant behaviors (e.g., posterior probabilities at 25% condition were lower than 0.5), they still increased with the proportion of norm-consistent behaviors. Meanwhile, when half or majority of group members exhibited norm-consistent behaviors, the posterior probabilities judged by participants were >0.5. These results indicated that participants can infer group norms based on the observed behaviors. Importantly, after controlling for gender and age, the main effect of the proportion of norm-consistent behaviors remained unchanged (*p* < 0.001). Neither gender, age, nor their interactions with the proportion of norm-consistent behaviors were significant (*p*s > 0.199), indicating that the pattern of results was not influenced by participants’ gender or age (see the Supplementary Materials for details).

**Fig. 11:**
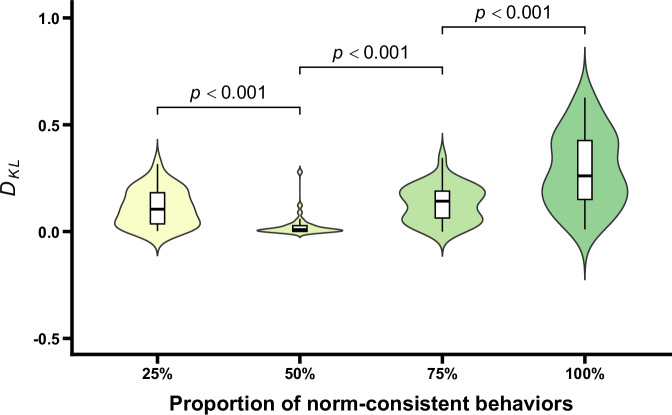
D_KL_ as a function of the proportion of norm-consistent behaviors. Violin plots and box plots are presented for each condition. Exact *p* values shown in the figure reflect comparisons between adjacent conditions.

The updating degrees of norm-related belief (i.e., *D*_*KL*_) under different conditions are shown in Fig. 11. One-way repeated measures ANOVA indicated that the main effect of the proportion of norm-consistent behaviors was significant, *F* (1.69, 52.47) = 41.03, *p* < 0.001, *η*_p_^2^ = 0.57. Further post hoc comparisons showed that *D*_*KL*_ at 50% norm-consistent behaviors was significantly lower than that at 25%, *t* (31) = −4.67, *p* < 0.001, Cohen’s *d* = −0.62. Conversely, *D*_*KL*_ at 75% norm-consistent behaviors was significantly higher than that at 50%, *t* (31) = 6.73, *p* < 0.001, Cohen’s *d* = 0.76, and *D*_*KL*_ at 100% was also significantly higher than that at 75%, *t* (31) = 7.28, *p* < 0.001, Cohen’s *d* = 1.16. One-sample *t* tests comparing *D*_*KL*_ in each condition with the value 0, indicating no updating in norm-related belief, showed that only *D*_*KL*_ at 50% of norm-consistent behaviors did not differ significantly from 0, *t* (31) = 2.88, *p* = 0.086. In contrast, *D*_*KL*_ in other conditions were significantly greater than 0, *t*s > 7.39, *p*s < 0.001, Cohen’s *d*s > 1.31. These results indicated that after observing the minority of group members exhibiting norm-consistent behaviors, participants showed increased updating of norm-related beliefs, with the judged probability of the existence of norms decreasing. When the majority of group members exhibited norm-consistent behaviors, participants’ norm-related beliefs were also updated, leading to a significant increase in the judged probabilities of existing norms. These results, along with those relating to the prior and posterior probabilities, indicated that participants make the inference of the existence of group norms based on the observed behaviors. Importantly, after controlling for gender and age, the main effect of the proportion of norm-consistent behaviors remained unchanged (*p* < 0.001). Neither gender, age, nor their interactions with the proportion of norm-consistent behaviors were significant (*p*s > 0.250), indicating that the observed pattern of results was not influenced by participants’ gender or age (see the Supplementary Materials for details).

### Experiment 3—predictions and comparisons of Bayesian network models

Moreover, by comparing the predictive performance of different Bayesian network models on participants’ judgments and identifying the best-performing model, the relationship between the inference of group norms and the inference of desires could be explored.

**Fig. 12:**
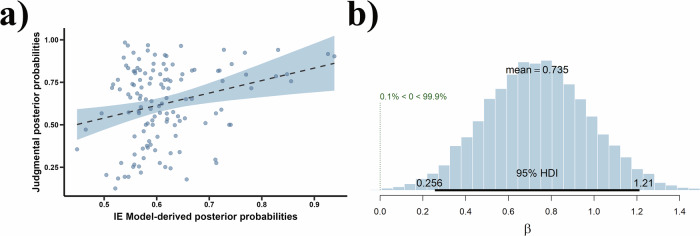
Results of the Bayesian hierarchical linear regression model for model-derived posterior probabilities predicting judgmental posterior probabilities. **a** The positive prediction effect of model-derived posterior probabilities on participants’ judgmental posterior probabilities. The blue region indicates the 95% confidence interval corresponding to *β*. **b** Posterior distribution of *β* drawn from the Bayesian hierarchical linear regression model. In this figure, the 95% HDI represents 95% highest density interval with the same meaning as the 95% CrI. The legend “0.1% < 0 < 99.9%” visually depicts the posterior probability mass for the slope parameter *β* that falls above or below zero, corresponding to *Pr* (*β* > 0) in the data.

The results of Bayesian hierarchical linear regression model showed that both the posterior probabilities derived from the IE model (*β* = 0.74, 95% CrI = [0.26, 1.21], *Pr* (*β* > 0) = 99.89%, *δ*_t_ = 18.76, as shown in Fig. 12) and the FC model (*β* = 0.50, 95% CrI = [0.06, 0.95], *Pr* (*β* > 0) = 98.69%, *δ*_t_ = 12.54) positively predicted the participants’ judgmental posterior probabilities. Furthermore, Rhat values equal to 1 indicate that both Bayesian hierarchical linear regressions model converged. However, data from the current experiment showed that the posterior probabilities derived from the DM model did not positively predict participants’ judgmental posterior probabilities, *β* = −0.18, 95% CrI = [−0.91, 0.55], *Pr* (*β* > 0) = 31.61%, *δ*_t_ = −3.93. The fact that both the IE and FC models positively predicted participants’ judgments indicates that group norm inferences were consistent with Bayesian updating regardless of the specific causal relationship between norms and desires.

Using leave-one-out cross-validation (LOO-CV) to compare the predictive performance of three Bayesian network models on participants’ judgments, the results are shown in Table 2. In Table 2, LOOIC is a model selection indicator that is similar to AIC or WAIC. The better the model prediction performance, the smaller the LOOIC. At the same time, ΔLOOIC represents the difference in LOOIC between each model and the best predictive model. The results showed that although both the IE model and the FC model positively predicted participants’ judgmental posterior probabilities, the LOOIC values indicated that the IE model had relatively better predictive performance. Therefore, given the relationship between the inference of norms and desires represented by the IE model, the current experiment suggested that the inference of group norms based on the behaviors of multiple group members was independent of the inference of desires.

**Table 2:** Predictive performance of different Bayesian network models

Model	LOOIC	Δ LOOIC
IE	−20.34	0.00
FC	−15.47	4.87
DM	−10.70	9.64

## Discussion

In this study, the computer-animated events were used as stimuli, and the number or proportion of group members exhibiting norm-consistent behaviors was manipulated to examine whether individuals infer group norms on the basis of the observed behaviors of group members and whether the inference is consistent with Bayesian updating. Given the diversity of group behavior, the present study also explored group norm inferences in the presence of norm-inconsistent deviant behaviors, which serve as negative evidence. Furthermore, the current study investigated whether the inference of group norms is independent of the inference of desires. The results showed that before the behaviors of group members were observed, participants’ judgmental probabilities of the existence of group norms (i.e., prior probabilities) did not significantly differ across conditions. However, after the behaviors of group members were observed, the judgmental probabilities of the existence of group norms (i.e., posterior probabilities) increased with the number or proportion of members exhibiting norm-consistent behaviors, and participants’ norm-related beliefs were significantly updated. Moreover, the inference of group norms is consistent with Bayesian updating. When the deviant behaviors inconsistent with norms were introduced, participants could still infer group norms on the basis of the observed behaviors of group members, showing differences in the prior probabilities judged by participants across conditions, while the judgmental posterior probabilities increased with the proportion of norm-consistent behaviors. Moreover, the updating degrees of norm-related beliefs increased significantly. These results indicated that the inference of group norms is robust. More importantly, the IE model, which represents the inference of group norms, is independent of the inference of desires, best predicted participants’ judgments, suggesting that the inference of group norms can be carried out independently.

With respect to the basis for the inference of group norms, the results obtained in Experiment 1 revealed that the norm-related prior probabilities judged by participants did not differ across conditions, whereas the judgmental posterior probabilities and the updating degrees of norm-related belief increased with the number of group members exhibiting norm-consistent behaviors. The results indicated that individuals can infer group norms on the basis of the observed behaviors of group members. Notably, several previous studies have preliminarily examined the basis for the inference of group norms and provided insights into understanding this issue^8,9^. However, in these studies, whether the inference of group norms occurs or not can be deduced only indirectly by comparing the behavioral changes before and after perceiving group norms. To be more specific, participants in these studies were usually asked to perform decision-making tasks, and their performance was influenced by other factors (e.g., group pressure). In contrast, in this study, the computer-animated events were used as stimuli, and participants were asked to directly judge the likelihood of the existence of group norms before and after the behaviors of group members were observed. This paradigm does not involve participants in decision-making tasks and enables a direct examination of the inference of group norms, providing more conclusive evidence for the basis of norm inferences. In addition, the present study primarily examines the inference of injunctive normative constraints within a novel group situation. Future research could further explore the inferential processes underlying other types of norms, such as descriptive norms, particularly in more complex social environments.

Furthermore, previous simulation studies suggested that the inference of group norms could be viewed as norm-related belief updating consistent with Bayesian Principles^12^. To investigate how the inference of group norms was made, the present study constructed a Bayesian inference model and examined the relationship between the model-derived posterior probabilities and participants’ judgmental posterior probabilities. The results revealed that there was a positive relationship between the two types of posterior probabilities, suggesting that the inference of group norms on the basis of the behaviors of group members aligns with Bayesian updating. In addition, to demonstrate that norm inference is characterized by a belief-updating process that integrates initial beliefs (priors) with subsequent observations, rather than being driven solely by observed evidence, a Frequency tracking model was introduced as a baseline for comparison with the Bayesian inference model. These two models diverge fundamentally in their computational approach: the Frequency tracking model relies exclusively on the cumulative tally of observed evidence, whereas the Bayesian inference model characterizes individuals as “lay Bayesians” who dynamically integrate new observations with prior beliefs. The comparative results reveal that while the Frequency tracking model can predict general behavioral trends, it fails to capture the finer-grained variability in participants’ norm-related judgments. In contrast, the Bayesian inference model provides a more precise approximation of norm inference. The superior predictive performance of the Bayesian inference model—particularly under rigorous statistical testing—underscores that group norm inference is a sophisticated belief-updating process rather than a mere accumulation of behavioral frequencies.

Notably, the results of Experiment 1 revealed that in the absence of deviant behaviors inconsistent with the norms, even observing a single group member who exhibited norm-consistent behaviors significantly increased participants’ updating degrees of norm-related beliefs. That is, when the number of norm-related behaviors was 1, participants’ judgmental posterior probability about the existence of norms was significantly >0.5. The results align with Tan and Ong’s^15^ findings, suggesting that although the consistent behaviors exhibited by multiple group members are key for individuals’ inference of group norms, under some conditions (i.e., the absence of deviant behaviors), a single instance of norm-consistent behaviors (i.e., weak evidence for the existence of norms) could trigger the inference of group norms. These results indicate that, similar to other Bayesian cognitive processes, the inference of group norms is highly sensitive to the norm-related evidence^36,37^.

On this basis, the present study further examined whether the inference of group norms can be carried out in the presence of deviant behaviors that are inconsistent with the norms. The results revealed that even with deviant behaviors, the probability of group norms being judged to exist increased with the proportion of norm-consistent behaviors, indicating that the inference of group norms could still be made. Moreover, the posterior probabilities derived from the Bayesian inference model positively predicted the participants’ judgmental posterior probabilities, suggesting that the inference of group norms aligns with Bayesian updating. On the one hand, the results provide further evidence that the consistent behavior exhibited by group members is an essential basis for the inference of group norms. On the other hand, the results also revealed that deviant behaviors in the group situation did not undermine the inference of group norms, demonstrating the robustness of the inference of group norms consistent with Bayesian updating^38^. These results may be because when group norms are inferred in a novel group situation, the core need of the individual is to exhibit adaptive behaviors to the current situation by observing the consistent behaviors of other group members^19^. Individuals focus on the external behavioral performance of other group members without overinterpreting the meanings underlying the deviant behaviors^20,21^. Thus, deviant behaviors are not treated as signals of individuals’ disagreement with the collective recognition of the group (such as group goals), and do not impair the belief about the group’s existence. Although the presence of deviant behaviors decreased participants’ judgmental probabilities about the existence of group norms, they did not disrupt the inference of group norms. Nevertheless, the boundary conditions under which observed behaviors are, or are not, taken as evidence for a norm cannot be determined in the current study. For instance, under conditions of causal opacity, similar behaviors may be more readily interpreted as evidence for norm inference^39^. Addressing this more complex issue requires further investigation in future research.

Finally, the current study explored the relationship between the inference of group norms and the inference of desires to test whether the norm inference is made independently. More specifically, considering the possible relationship between the inference of group norms and the inference of desires, the current study constructed the Independent Effect (IE) model, the Fully Connected (FC) model, and the Desire Mediation (DM) model. The results showed that the IE model better predicted participants’ judgments, indicating that individuals make the inference of group norms independently of the inference of desires in a novel situation. Consistent with previous findings, the results provide evidence, in part, for the existence of shared intentionality, suggesting that humans have the unique ability to directly recognize the shared mental states of group members^26^.

However, when the relationship between two types of inferences was explored, Tan and Ong^15^ reported that individuals’ inference of group norms was grounded in the inference of desire. These inconsistent results may be attributed to two reasons. First, in Tan and Ong’s^15^ study, the researchers did not intentionally simulate a group situation, and only the behavior of a single individual was presented in the experiment. In such cases, it is difficult for individuals to identify the cause of behavior performed by other members (i.e., to comply with group norms or to satisfy their desires), making it impossible to directly infer group norms on the basis of the observed behaviors. Rather, the present study simulated a group situation through computer-animated events, which provided a possibility for separating the influence of group norms and desires on behavioral performance. The results showed that the inference of group norms was conducted independently of the inference of desire. Second, the norms involved in Tan and Ong’s^15^ study are common in daily lives (i.e., not littering in public); thus, eliminating the effects of participants’ experiences and familiarity on the inference of norms is difficult. In particular, participants are frequently exposed to these norms in their daily lives, which changes their desires through norm internalization^25^. That is, after norms were internalized, participants may believe that group members wanted to perform behaviors consistent with these norms; thus, participants’ inferences of group norms were based on the inference of desires. In contrast, the current study presented participants with novel groups and norms, controlling the impact of participants’ prior experience and familiarity.

Although a negative slope was observed when the DM model was used to predict participants’ judgments, the statistical evidence supporting this result was insufficient. The inability of the DM model to predict participants’ judgments suggests that their inferences of norms and desires remained separate, indicating that the norms were not internalized—likely because brief exposure to novel norms was insufficient to induce internalization. Future research could investigate whether repeated exposure to a novel norm strengthens the connection between desire and norm inferences, potentially aligning DM model predictions with participants’ judgments. Additionally, future experimental designs could directly disentangle the influence of desires, providing a clearer understanding of the relationship between norm and desire inferences.

In conclusion, using computer-animated events as stimuli and manipulating the number or proportion of norm-consistent behaviors, this study examined how individuals infer group norms. Furthermore, by introducing deviant behaviors that are inconsistent with norms and constructing different Bayesian network models to reflect the relationships between group norm inferences and personal desire inferences, this study investigated the robustness and independence of group norm inferences. The results demonstrated that individuals can infer group norms on the basis of the observed behaviors of group members, and the inference aligns with Bayesian updating, demonstrating robustness and independence from the inference of personal desires.

## Methods

Experiment 1 investigated the basis of group norm inferences using computer-animated events. To measure participants’ inherently subjective norm-related beliefs^40–42^, a well-established methodology in social and cognitive psychology was adopted^43–45^. This approach involves asking participants to judge the probabilities of the existence of group norms before and after they observe varying numbers of norm-consistent behaviors, providing prior and posterior probabilities, and has been widely used in previous studies to measure subjective beliefs related to cooperation^46^, fairness^47^, and even norms^15^. In addition, the updating degrees of belief related to group norms were calculated on the basis of these probabilities. Furthermore, Experiment 1 also aimed to clarify the computational account of group norm inferences by constructing a Bayesian inference model and examining whether participants’ judgments could be predicted by the model’s output.

Experiment 1 aimed to demonstrate that people can infer group norms on the basis of the observed behaviors of group members, and this inference aligns with Bayesian updating. Given the diversity of group behavior, Experiment 2 introduced deviant behaviors inconsistent with the norms to further explore norm inference in the presence of negative evidence.

In Experiment 3, the relationship between group norm inferences and desire inferences was explored. Specifically, the measurements related to inference of desires were introduced, and different Bayesian network models were constructed. Afterwards, whether the inference of group norms is independent of the inference of desires was explored through comparing the predictive performance of different models on participants’ judgmental posterior probabilities.

### Participants

Thirty-two paid volunteers (14 males and 18 females) participated in Experiment 1. The age range of the participants was 19–26 years (*M* = 22.43 years, *SD* = 2.24 years). All participants were right-handed and had normal or corrected-to-normal vision and no history of neurological disorders or family history of mental disorders. Before the experiment, all the participants were fully notified of the experimental requirements and signed informed consent forms. This study was approved by the Ethics Committee of East China Normal University (HR2-0004-2021).

In Experiment 2, the same recruitment criteria as in Experiment 1 applied. Thirty-two paid volunteers (14 males, 18 females) participated, with ages ranging from 18 to 27 years (*M* = 21.31 years, *SD* = 2.46 years).

In Experiment 3, the same criteria were followed, with 32 paid volunteers (12 males, 20 females) aged 18 to 29 years (*M* = 21.75 years, *SD* = 2.77 years).

The sample size for this study was determined using G*Power 3.1 software^48^. Given the absence of prior research with a comparable experimental design, a conventional value for a medium effect size, *f* = 0.25, was chosen^49^. This approach is a standard practice when no specific effect size is available from the literature^50^. On the basis of the specific design of this experiment and the alpha value set at 0.05, a priori power analysis revealed that a sample size of more than 30 participants would achieve a statistical power greater than 0.90. Furthermore, a sensitivity power analysis confirmed that the sample size of the current study (*N* = 32) is sufficient to detect effect sizes as small as *f* = 0.24 with a power of 0.90. To provide a comprehensive view of these power considerations, a sensitivity curve illustrating the relationship between effect size and statistical power given our sample size is provided in the Supplementary Materials (see Supplementary Fig. 1).

### Experimental design

Experiment 1 used computer-animated events, featuring five cartoonized agents as stimuli. Four of these agents could exhibit norm-consistent behaviors, while one served as the target agent. Following the principle that individuals often follow specific behavior patterns to comply with group norms^51^, two potential norm types were defined: agents moving toward the bottom corners of the screen either in a straight-line movement or in a jumping movement. The movement direction (e.g., left or right) was not part of the norm itself but served as a controlled, balanced factor within the experimental design. Consistent with previous work^52^, the straight-line movements of agents approaching the bottom corners were considered norm-consistent behaviors.

Experiment 1 employed a one-way within-participants design, with the independent variable being the number of norm-consistent behaviors (1 vs. 2 vs. 3 vs. 4). Specifically, when the number of norm-consistent behaviors was 1, only one agent exhibited norm-consistent behavior while the other three remained static. The other levels followed the same pattern. Participants’ judgments were always made regarding the behavior of the designated target agent or the situation in which it was located.

The dependent variables in Experiment 1 are the prior and posterior probabilities related to norms, as well as the updating degrees of norm-related belief. The prior and posterior probabilities are participants’ judgments (on a scale of 0% to 100%, where 0% indicates “definitely not exist” and 100% indicates “definitely exists”) regarding the likelihood of the existence of norms in the situation where the target agent was located, before and after observing the behaviors of the group, respectively. Moreover, the updating degrees of belief corresponding to the prior and posterior probabilities are measured by Kullback–Leibler Divergence (*D*_*KL*_). The larger this value is, the greater the updating degree of belief. The specific calculation method for *D*_*KL*_ is detailed in the data analysis section.

The design of Experiment 2 was similar to that of Experiment 1, with several differences. Specifically, to investigate how deviant behaviors inconsistent with norms influence the inference of group norms, all four target agents moved during the presentation of norm-consistent behaviors. They could exhibit either the norm-consistent behaviors (i.e., straight-line movements within a specific area at the bottom of the screen) or norm-inconsistent behaviors (i.e., jumping movements within a specific area at the bottom of the screen). By varying the number of group members exhibiting deviant behaviors, the proportion of norm-consistent behaviors was manipulated. Therefore, Experiment 2 employed a within-participants design with the independent variables being the proportion of norm-consistent behaviors (25% vs. 50% vs. 75% vs. 100%). Additionally, the number of trials in this experiment was increased to 48.

Experiment 3 followed a similar design to Experiment 2, with additional modifications. In particular, to construct different Bayesian network models, measurements of desire-related prior probabilities and posterior probabilities, as well as the conditional probabilities necessary for each model, were introduced. Participants’ desire-related probabilities were quantified through direct, subjective judgments. Before and after observing the behaviors of group members, participants were asked to judge the probabilities that the target agent wanted to exhibit norm-consistent behaviors. Ratings were made on a slider ranging from 0% to 100%, yielding measures of both desire-related prior and posterior probabilities (see Supplementary Table 2 for details).

### Experimental apparatus and stimuli

The stimuli in all three experiments were presented on a gray background using a 24-inch Liquid Crystal Display (LCD) monitor (1024 × 768-pixel resolution and 60 Hz refresh rate). The participants sat approximately 60 cm away from the screen. Referring to the methods in previous studies^33,53^, the cartoon characters were also composed of usual shapes (such as triangles, squares, circles, etc.) and colors (such as red [RGB: 255, 0, 0], yellow [RGB: 255, 255, 0], blue [RGB: 0, 0, 255], purple [RGB: 96, 25, 134], etc.). By combining different shapes and colors, 100 unique cartoon characters were created. Examples can be found on the OSF platform: https://osf.io/xtuz9/?view_only=b1a876617e9b46e9bb09bc11569cde4e. In each experiment, a cartoon character was randomly selected for each trial. Participants were informed that the cartoon characters presented in different trials were independent of each other and belonged to different groups.

### Experimental procedure

Experiment 1 used custom software written in MATLAB using the Psychophysics Toolbox library^54^. This experiment consisted of 32 trials, with 8 trials in each of the 4 conditions (the number of norm-consistent behaviors: 1 vs. 2 vs. 3 vs. 4), and these trials were presented randomly throughout the experiment. Prior to the main experiment, participants were informed that their objective was to judge questions related to group norms based on the observed behaviors of agents. They then completed practice trials to ensure familiarity with the procedural flow and slider operations. Crucially, these practice trials were independent of the main experiment and served only to ensure task comprehension. To prevent potential bias, all instructions remained strictly neutral, including the explicit statement that there were no “correct” or “incorrect” answers. The main experiment commenced only after participants had completed the practice phase and confirmed that they had no remaining questions regarding the task or any other procedural aspects.

**Fig. 13:**
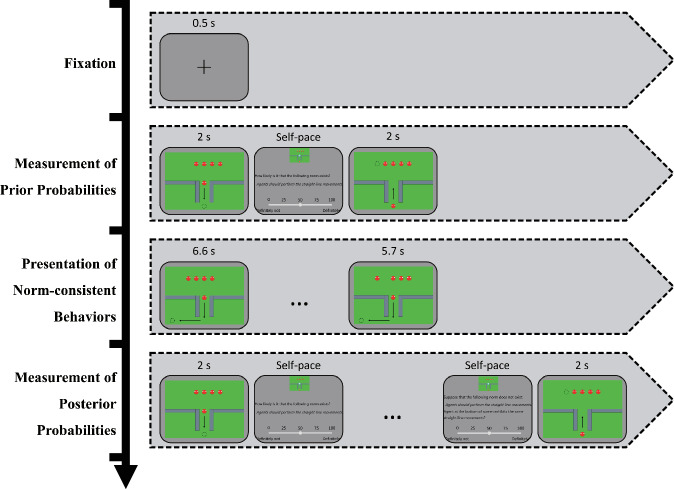
Representative trial sequences from Experiment 1. Each trial consisted of three stages: measurement of prior probabilities, presentation of norm-consistent behaviors and measurement of posterior probabilities. A fixation cross was presented for 500 ms at the beginning of each trial. Durations of the corresponding screens are indicated above each display, with judgment screens being self-paced.

To investigate the basis of group norm inferences, participants were required to judge the likelihood of the existence of group norms before and after observing varying numbers of group members performing norm-consistent behaviors (i.e., prior and posterior probabilities). Therefore, each trial in this experiment could be subdivided into three stages: measurement of prior probabilities, presentation of norm-consistent behaviors and measurement of posterior probabilities (Fig. 13). Specifically, at the beginning of each trial, a fixation cross was presented at the center of the screen for 500 ms, followed by the three stages in sequence as described below.

During the measurement of prior probabilities, those related to norms were measured. Participants were required to judge the likelihood of the existence of norms before observing the behaviors of group members. The test questions were related to the target agents or the situation in which they were placed. Specifically, during this stage, five identical agents were presented at the top center of the screen, helping participants construct the group representation through perceptual cues such as spatial proximity and similarity^55,56^. The target agent, located at the far left or far right, did not display any norm-related behaviors during the experiment and served only as the object of judgment. The position of the target agent (far left or far right) was random in each trial and balanced throughout the experiment. Once the target agent moved to the bottom center of the screen (i.e., the testing position), a question related to the prior probabilities (see Supplementary Table 1 for details) appeared at the middle of the screen. Participants were requested to adjust the slider at the bottom of the screen using the “F” and “J” keys to select a value, ranging from 0% to 100%. After adjusting the slider, the participants needed to press the space key to confirm their judgments. Afterward, the target agent returned to the initial position, marking the end of this stage.

During the presentation of norm-consistent behaviors, the norm-related behaviors of group members were presented. The agents (i.e., the sample agents) opposite the target agent began to move in sequence, and they would exhibit straight-line movements (i.e., the norm-consistent behaviors) within a specific area at the bottom of the screen. In different trials, the numbers of sample agents moving depended on the experimental conditions. For instance, if the number of norm-consistent behaviors was 1, one sample agent would move while the remaining three agents remained still. However, if the number was 2, the two sample agents would move sequentially, and the remaining 2 agents would remain static. The other conditions followed the same pattern. The straight-line movements, as a norm, randomly approached the left or right side at the bottom of the screen, and the approaching sides were balanced throughout the experiment. Once all the sample agents in the current condition had finished moving, this stage ended.

The measurement of posterior probabilities focused on norm-related posterior probabilities and the different kinds of conditional probabilities necessary for the construction of the Bayesian inference model. More specifically, after observing the behaviors of multiple group members, the target agent moved to the testing position. The participants were subsequently asked to judge the likelihood of the existence of norms (i.e., the posterior probability) and the likelihood of observing the norm-consistent behaviors of group members under the assumption of the existence or absence of norms (i.e., the conditional probabilities required by the Bayesian inference model).

The procedures in Experiments 2 and 3 were identical to those in Experiment 1, with adjustments to the test questions and the presented behaviors to match each experiment’s specific design. Animations illustrating the experimental flow can be found on the OSF platform: https://osf.io/xtuz9/?view_only=b1a876617e9b46e9bb09bc11569cde4e.

### Data Analysis

To explore whether people can infer norms on the basis of the observed behaviors of multiple group members, Experiment 1 compared the prior probabilities (i.e., the likelihood of the existence of norms before observing the behaviors), the posterior probabilities (i.e., the likelihood of the existence of norms after observing the behaviors), and the updating degrees of belief related to the prior and posterior probabilities (i.e., *D*_*KL*_) under different conditions. Moreover, to examine whether the inference of norms aligns with Bayesian updating, Experiment 1 further explored whether participants’ posterior probability judgments were predicted by the posterior probabilities derived from the model.

Using the prior and posterior probabilities as dependent variables, a one-way repeated measures analysis of variance (ANOVA) was conducted to analyze the effects of the number of norm-consistent behaviors. ANOVA in this experiment was performed using the *bruceR* package^57^ in R (version 4.2.2; R Core Team, 2022). In addition, building on the ANOVA, age was included as a covariate and gender was included as a between-subjects factor to confirm that these effects were not influenced by demographic variables (the same procedure was applied in the subsequent experiments).

Experiment 1 further compared the updating degrees of norm-related beliefs under different conditions. By calculating the Kullback–Leibler Divergence (*D*_*KL*_) upon the probability distributions, the updating degrees of norm-related beliefs can be measured^58,59^. Therefore, this experiment also used *D*_*KL*_ to index the belief updating, capturing overall changes in the distribution rather than just the numeric difference between the prior and posterior probabilities. Specifically, Experiment 1 represented participants’ norm-related beliefs as discrete probability distributions. Building on this, *D*_*KL*_ was calculated for each participant under all conditions using Eq. 1. One-way repeated-measures ANOVA was subsequently conducted to examine the effect of the number of norm-consistent behaviors on *D*_*KL*_. In addition, building on this ANOVA, age was also included as a covariate and gender as a between-subjects factor to ensure that the effects were not influenced by demographic variables (the same procedure was applied in the subsequent experiments).

1 $${D}_{{KL}}=\mathop{\sum }\limits_{i=1}^{N}p\left({x}_{i}\right)* (\log p\left({x}_{i}\right)-\log q\left({x}_{i}\right))$$

In Eq. 1, $p\left({x}_{i}\right)$ denotes the posterior beliefs related to norms held by participants, which can be divided into the probabilities of norms existing and not existing as perceived by participants. Moreover, $q\left({x}_{i}\right)$ indicates the prior beliefs related to the norms held by participants, which can also be divided into the same two parts.

In Experiment 1, the posterior probabilities of the Bayesian inference model were calculated, and whether they predicted the posterior probabilities judged by participants was explored by hierarchical linear regression analysis. Moreover, the posterior probabilities of the Bayesian inference model under different conditions were calculated through Eq. 2:

2 $$P\left(N/A\right)=\frac{P\left(N\right)* P\left({A|N}\right)}{P\left(N\right)* P\left({A|N}\right)+P\left({\neg N}\right)* P\left({A|}\neg N\right)}$$

In Eq. 2, P (*N*) denotes the prior probabilities of the existence of group norms. *P* (¬*N*) represents the prior probability of the absence of norms. *P* (*A*│*N*) denotes the probability of the sample agents exhibiting the observed behaviors given the existence of norms. *P* (*A*│¬*N*) represents the probability of the sample agents displaying the observed behaviors when the norms do not exist (see Supplementary Table 1 for details). In addition to the priors, these conditional probabilities were measured and used as inputs in Eq. 2 to derive the posterior probabilities in the Bayesian inference model.

To explore the prediction of model-derived posterior probabilities on participants’ judgmental posterior probabilities, in Experiment 1, a Bayesian hierarchical linear regression model was conducted^60^. Given that the number of norm-consistent behaviors is a within-participant variable, the derived posterior probabilities and participants’ judgmental posterior probabilities under different conditions for the same participants are repeated measures data. Compared with ordinary linear regression, Bayesian hierarchical linear regression, which takes into account different levels of variability, is more suitable for repeated measures data^61^. Thus, the analysis was used in Experiment 1.

The *brms* package in R^62^ was used for the Bayesian hierarchical linear regression model. The model included a random intercept and slope for each participant, a widely adopted approach for such designs^63^. The regression equations were constructed with the model-derived posterior probabilities under different conditions as the independent variables and the participants’ judgmental posterior probabilities under different conditions as the dependent variable. Following previous research^61^, the default weakly informative Gaussian priors from the *brms* package were adopted. The Markov chain Monte Carlo (MCMC) algorithm, with 2 chains and 10^4^ iterations (the first 2000 iterations were used to calibrate the MCMC, i.e., warm-up iterations) for each chain, was applied to obtain the posterior distribution for each parameter (for details, see McElreath, 2020^64^). The effect size *δ*_t_ was calculated by dividing the estimated difference between group means by the square root of the sum of all variance components in the model, which is consistent with previous research^61^.

By conducting a Bayesian hierarchical linear regression model to explore whether the model-derived posterior probabilities predict the participants’ judgmental posterior probabilities, the slope *β* and its 95% credible interval (95% CrI), drawn from the posterior distribution, were reported according to previous studies^61,65,66^. The probability of *β* being greater than 0, i.e., *Pr* (*β* > 0), was also reported. If the corresponding 95% CrI does not include 0, or if the probability of *β* being greater than 0 exceeds 95%, it indicates that participants’ judgmental posterior probabilities can be predicted by the model-derived posterior probabilities. Additionally, the effect sizes corresponding to the slope *β* (i.e., *δ*_t_) were reported, with larger values indicating larger effect sizes^61,67^. Finally, Rhat values were close to 1, suggesting that the Bayesian hierarchical linear regression model has converged^68–70^.

Moreover, the central argument of the current study is that norm inference is a Bayesian belief updating that integrates both initial beliefs (priors) and subsequent observations. To address this claim, and in light of evidence that tracking the frequency of observed evidence has been shown to enable complex cognitive inferences^71^, a Frequency tracking model was constructed (see the Supplementary Materials for details) drawing on previous research^72,73^. This model, which relies solely on observed behaviors, served as a baseline model to assess whether the Bayesian inference model better captures norm inference. The predictive performance of the Frequency tracking model for participants’ judgments was also examined using a Bayesian hierarchical linear regression. Notably, the model outputs were checked to assess which model could better capture the nuanced variability in participants’ responses^71^. Furthermore, by incorporating diverse random effects into the hierarchical regression to account for various sources of variability, the analysis was conducted to determine which model could provide a more nuanced predictive performance.

Building on this framework, Experiment 2 examined norm inference in the presence of deviant behaviors. The independent variable was the proportion of norm-consistent behaviors, and the data were analyzed using the same approach as in Experiment 1. Specifically, to explore whether people make the inference of group norms based on the observed behaviors of group members in the presence of deviant behaviors as negative evidence, the present experiment compared the prior probabilities, the posterior probabilities, and the updating degrees of norm-related belief across different conditions. Furthermore, to examine whether the inference of group norms with negative evidence was still performed consistently with Bayesian updating, the Bayesian inference model was constructed, and then the relationship between the model-derived posterior probabilities (calculated using Eq. 2, as in Experiment 1) and the participants’ judgmental posterior probabilities was explored. In addition, a Frequency tracking model was built as a baseline to assess whether the Bayesian inference model, integrating prior beliefs with subsequent observations, better captures norm inference (see the Supplementary Materials for details). The analytic tools used in this experiment were the same as in Experiment 1.

To confirm that people make the inference of group norms based on observed behaviors, Experiment 3 compared prior probabilities, posterior probabilities, and the updating degrees of norm-related belief (i.e., *D*_*KL*_) across conditions, using the same analysis method and tools as Experiment 2. Desire-related prior and posterior probabilities were analyzed similarly to norm-related prior and posterior probabilities, with results provided in the Supplementary Materials.

**Fig. 14:**
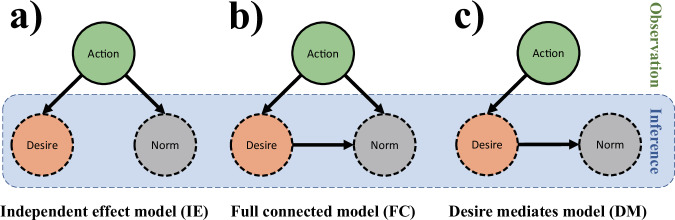
Possible relationships between the inference of group norms and the inference of desires. **a** Representation for inference of group norms independent of inference of desires. **b** Representation for inference of group norms independent of or grounded on inference of desires. **c** Representation for inference of group norms grounded in inference of desires.

Furthermore, to explore the relationship between the inference of group norms and the inference of desires^28–30^, and following the methodology of Tan and Ong^15^, three Bayesian network models were constructed: the IE Model, the Full Connected (FC) Model, and the Desire Mediation (DM) Model. The models differ in their assumptions about how norms, desires, and actions are causally related: the IE model treats norms and desires as independent influences on actions, the FC model assumes norms influence desires and both jointly affect actions, and the DM model assumes norms influence actions entirely through desires (see Fig. 14 for a graphical illustration and the Supplementary Materials for computational details). Each model requires distinct conditional probabilities (see Supplementary Table 2), which were measured to generate the model-derived posterior probabilities. A Bayesian hierarchical linear regression model was used to assess the relationship between model-derived posterior probabilities and participants’ judgmental posterior probabilities, with the posterior probabilities from the models as independent variables and the participants’ judgments as the dependent variable.

Moreover, the predictive performance of three Bayesian network models on participants’ responses was evaluated using LOO-CV^74^, which effectively assesses out-of-sample predictive performance of models^64^. The LOO information criterion (LOOIC) was reported for each model, serving as an indicator similar to the Akaike Information Criterion (AIC)^75^ or Watanabe-Akaike Information Criterion (WAIC)^76^. Lower LOOIC values indicate better model predictive performance^60,77^.

The construction of Bayesian network models and the calculation of model-derived posterior probabilities were performed using the *bnlearn* package in R^78^. The other tools used in this experiment was same as in Experiments 1 and 2.

For all experiments, Bonferroni-adjusted *p* values were reported when multiple comparisons were involved. Adjusted *p* values were calculated by multiplying the unadjusted *p* values by the number of comparisons and capping the resulting values at 1.00, allowing direct comparison with the conventional α = 0.05 threshold^79–82^. Moreover, *p* values and degrees of freedom were Greenhouse-Geisser corrected if the sphericity assumption was violated.

## Supplementary information

Supplementary materials

## Data Availability

All data and example trial videos for each condition have been made publicly available via the Open Science Framework and can be accessed at https://osf.io/xtuz9/?view_only=b1a876617e9b46e9bb09bc11569cde4e.

## Code availability

Analyses were conducted using R version 4.2.2. All code necessary to replicate the results of this study is publicly available on the Open Science Framework at https://osf.io/xtuz9/?view_only=b1a876617e9b46e9bb09bc11569cde4e.
