# Supplementary material for: A Bayesian perspective on observers’ inference of group norms
Source: NPJ Sci Learn. 2026 Mar 2;11:24. doi: 10.1038/s41539-026-00405-x (PMC13065979; doi:10.1038/s41539-026-00405-x)
Supplement: Supplementary file 1 — Supplementary materials [file 41539_2026_405_MOESM1_ESM.pdf]

Supplementary Materials

1 Measurements for different probabilities in Experiments 1 and 3

Supplementary Table 1. Measurements for different probabilities in Experiments 1

Target items	Measurements
Norm-related prior probabilities, P (N)	In the animated events, agents can perform either straight-line or jumping movements. Given this, how likely is it that the following norm exists in this situation? *Agents should perform the straight-line movements.*
Norm-related posterior probabilities, P (N | A)	Suppose that agents at the bottom of screen exhibited the same straight-line movements as the previous X agents. Given this, how likely is it that the following norm exists in this situation? *Agents should perform the straight-line movements.*
Probability that the observed behaviors occur in the existence of group norms, P (A | N)	In the animated events, agents can perform either straight-line or jumping movements. Suppose that the following norm exists in this situation: *Agents should perform the straight-line movements.* Given this, how likely would it be that agent at the bottom

	of screen exhibits the same straight-line movements as the previous *X* agents?
Probability that the observed behaviors occur in the absence of group norms, P (A | ¬ N)	In the animated events, agents can perform either straight-line or jumping movements. Suppose that the following norm does not exist in this situation: *Agents should perform the straight-line movements.* Given this, how likely would it be that agent at the bottom of screen exhibits the same straight-line movements as the previous *X* agents?

Note. *X* will be replaced with different values depending on the specific conditions.

Supplementary Table 2. Measurements for different probabilities in Experiments 3

Target items	Measurements
Norm-related prior probabilities, P (N)	In the animated events, agents can perform either straight-line or jumping movements. Given this, how likely is it that the following norm exists in this situation? *Agents should perform the straight-line movements.*
Desire-related prior	In the animated events, agents can perform either straight-line or jumping movements. Given this, how likely is it that

probabilities, $P(D)$	the following fact exists in this situation?<br>*The agent at the bottom of screen wants to perform the straight-line movements.*
Probability that the agent wants to perform norm-consistent behaviors in the existence of group norms, $P(D \mid N)$	In the animated events, agents can perform either straight-line or jumping movements. Suppose that the following norm exists in this situation:<br>*Agents should perform the straight-line movements.*<br>Given this, how likely is it that agent at the bottom of screen wants to perform the straight-line movements?
Probability that the agent wants to perform norm-consistent behaviors in the absence of group norms, $P(D \mid \neg N)$	In the animated events, agents can perform either straight-line or jumping movements. Suppose that the following norm does not exist in this situation：<br>*Agents should perform the straight-line movements.*<br>Given this, how likely is it that agent at the bottom of screen wants to perform the straight-line movements?
Norm-related posterior probabilities, $P(N \mid A)$	Suppose that agent at the bottom of screen exhibited the same straight-line movements as the previous *X* agents. Given this, how likely is it that the following norm exists in

	this situation?<br><br>*Agents should perform the straight-line movements.*
Desire-related posterior probabilities, $P\ (D \mid A)$	Suppose that agent at the bottom of screen exhibited the same straight-line movements as the previous *X* agents. Given this, how likely is it that the following fact exists in this situation?<br><br>*The Agent wants to perform the straight-line movements.*
Probability that the observed behaviors occur in the existence of group norms, $P\ (A \mid N)$	In the animated events, agents can perform either straight-line or jumping movements. Suppose that the following norm exists in this situation:<br><br>*Agents should perform the straight-line movements.*<br><br>Given this, how likely would it be that agent at the bottom of screen exhibits the same straight-line movements as the previous *X* agents?
Probability that the observed behaviors occur in the absence of group norms, $P\ (A \mid \neg N)$	In the animated events, agents can perform either straight-line or jumping movements. Suppose that the following norm does not exist in this situation:<br><br>*Agents should perform the straight-line movements.*<br><br>Given this, how likely would it be that agent at the bottom

	of screen exhibits the same straight-line movements as the previous *X* agents?
Probability that the observed behaviors occur when the agent wants to perform norm-consistent behaviors, $P\ (A \mid D)$	In the animated events, agents can perform either straight-line or jumping movements. Suppose that the following fact exists in this situation: *The agent at the bottom of screen wants to perform the straight-line movements.* Given this, how likely would it be that agent at the bottom of screen exhibits the same straight-line movements as the previous *X* agents?
Probability that the observed behaviors occur when the agent does not want to perform norm-consistent behaviors, $P\ (A \mid \neg D)$	In the animated events, agents can perform either straight-line or jumping movements. Suppose that the following fact does not exist in this situation: *The agent at the bottom of screen wants to perform the straight-line movements.* Given this, how likely would it be that agent at the bottom of screen exhibits the same straight-line movements as the previous *X* agents?
Probability that the	In the animated events, agents can perform either straight-

observed behaviors occur in the existence of group norms and when agent wants to perform norm-consistent behaviors, $P\ (A \mid N, D)$	line or jumping movements. Suppose that the following norm exists in this situation:<br>*Agents should perform the straight-line movements.*<br>And the following fact exists in this situation：<br>*The agent at the bottom of screen wants to perform the straight-line movements.*<br>Given this, how likely would it be that agent at the bottom of screen exhibits the same straight-line movements as the previous *X* agents?
Probability that the observed behaviors occur in the existence of group norms and when agent does not want to perform norm-consistent behaviors, $P\ (A \mid N, \neg D)$	In the animated events, agents can perform either straight-line or jumping movements. Suppose that the following norm exists in this situation:<br>*Agents should perform the straight-line movements.*<br>And the following fact does not exist in this situation:<br>*The agent at the bottom of screen wants to perform the straight-line movements.*<br>Given this, how likely would it be that agent at the bottom of screen exhibits the same straight-line movements as the previous *X* agents?

Probability that the observed behaviors occur in the absence of group norms and when agent wants to perform norm-consistent behaviors, $P(A \mid \neg N, D)$	In the animated events, agents can perform either straight-line or jumping movements. Suppose that the following norm does not exist in this situation:<br>*Agents should perform the straight-line movements.*<br>And the following fact exists in this situation:<br>*The agent at the bottom of screen wants to perform the straight-line movements.*<br>Given this, how likely would it be that agent at the bottom of screen exhibits the same straight-line movements as the previous *X* agents?
Probability that the observed behaviors occur in the absence of group norms and when agent does not want to perform norm-consistent behaviors, $P(A \mid \neg N, \neg D)$	In the animated events, agents can perform either straight-line or jumping movements. Suppose that the following norm does not exist in this situation:<br>*Agents should perform the straight-line movements.*<br>And the following fact does not exist in this situation:<br>*The agent at the bottom of screen wants to perform the straight-line movements.*<br>Given this, how likely would it be that agent at the bottom of screen exhibits the same straight-line movements as the

	previous X agents?

Note. X will be replaced with different values depending on the specific conditions.

2 Sensitivity Power Analysis

Given that sample size calculations are highly sensitive to the choice of effect size, a sensitivity power analysis was conducted to provide a comprehensive understanding of the statistical power considerations in this study. Unlike an a priori power analysis, which estimates the required sample size for a specific effect size, a sensitivity analysis determines the minimum effect size that can be reliably detected given the fixed sample size ($N = 32$) and the significance level ($\alpha = 0.05$).

As shown in Supplementary Figure 1, the relationship between statistical power and effect size (f) was examined across a plausible range. The results indicated that with the current sample size of $N = 32$ and a power level of 0.90, the study is sufficiently sensitive to detect effect sizes as small as $f = 0.24$.

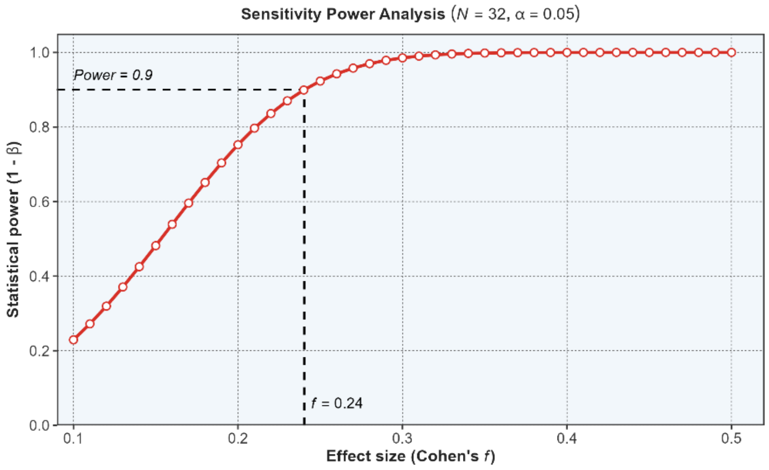

Supplementary Figure 1. Statistical power as a function of effect size (*f*) for a fixed sample size (*N* = 32).

These findings suggest that the study was sufficiently powered to detect medium-sized effects, conventionally benchmarked at $f = 0.25$, indicating that the sample size was appropriate for the experimental design.

3 Frequency tracking model construction and comparison

Specifically, to construct a model reflecting how norm inference is influenced solely by observed behaviors, the Frequency tracking model was constructed based on the frequency of members who exhibited norm-consistent behaviors and those who did not. The specific models for Experiment 1 and Experiment 2 are as follows:

$$P = \frac{N_A^{\theta}}{N_{\mathrm{A}}^{\theta} + N_{\neg\mathrm{A}}^{\gamma}} \qquad \text{(Experiment 1)}$$

$$P = \frac{N_A^{\theta}}{N_{\mathrm{A}}^{\theta} + N_{\neg\mathrm{A}}^{\theta}} \qquad \text{(Experiment 2)}$$

Where N_{A} represents the number of members who exhibited norm-consistent behaviors, and $N_{\neg\mathrm{A}}$ represents the number of members who did not. The parameter θ reflects the influence of behavioral frequency on norm inference, with a larger value indicating a greater influence. It is worth noting that in Experiment 1, the group members who did not exhibit norm-consistent behaviors remained stationary. This could have a different influence on norm inference compared to a situation where they exhibit a specific deviant behavior (Albarracín et al., 2019). To represent this distinction, the parameter γ was introduced in the model for Experiment 1.

The Frequency tracking models were fitted using the Hamiltonian Monte Carlo engine Stan in R v.4.4.1 from Rstan v.2.32.7. Four Markov chain Monte Carlo

(MCMC) chains were run for 4000 iterations, with the initial 2000 iterations designated as a warm-up period. We used weakly informative normal priors centered on 0 for θ and γ parameters. A fixed random seed was also used for reproducibility. For all analyses, visual inspection of trace plots and rank histograms suggested good model convergence and no problematic autocorrelation, with convergence confirmed by the Gelman-Rubin criterion $R \leq 1.01$.

After determining the optimal values for θ or γ, the prediction for participants' judgments of norm existence probability was calculated using the Frequency tracking model. Then a Bayesian hierarchical linear regression was applied to examine the predictive performance of the model's output on participants' actual judgments. The results showed that both the Bayesian inference model (Experiment 1: $\beta = 0.69$, 95% CrI = [0.29, 1.15], *Pr* ($\beta > 0$) = 100.00%, $\delta_t = 8.31$; Experiment 2: $\beta = 0.87$, 95% CrI = [0.50, 1.25], *Pr* ($\beta > 0$) = 100.00%, $\delta_t = 16.50$) and the Frequency tracking model (Experiment 1: $\beta = 0.48$, 95% CrI = [0.38, 0.58], *Pr* ($\beta > 0$) = 100.00%, $\delta_t = 1.98$; Experiment 2: $\beta = 0.78$, 95% CrI = [0.70, 0.86], *Pr* ($\beta > 0$) = 100.00%, $\delta_t = 3.69$) could positively predict participants' actual judgments.

However, following the model evaluation strategies of prior research (Baker et al., 2025), a careful examination revealed that the predictive output of the Frequency tracking model was relatively coarse. The Frequency tracking model produced only a small number of fixed estimates, causing the data to cluster into distinct groups (as shown in Supplementary Figure 2b and 2d). This pattern indicates that the Frequency tracking model treats data points within these clusters as indistinguishable, although

participants' actual responses varied within them. In contrast, the Bayesian inference model generated a continuous range of estimates, which suggests it was able to capture the more detailed variability in participants' responses—a nuance missed by the Frequency tracking model (as shown in Supplementary Figure 2a and 2c).

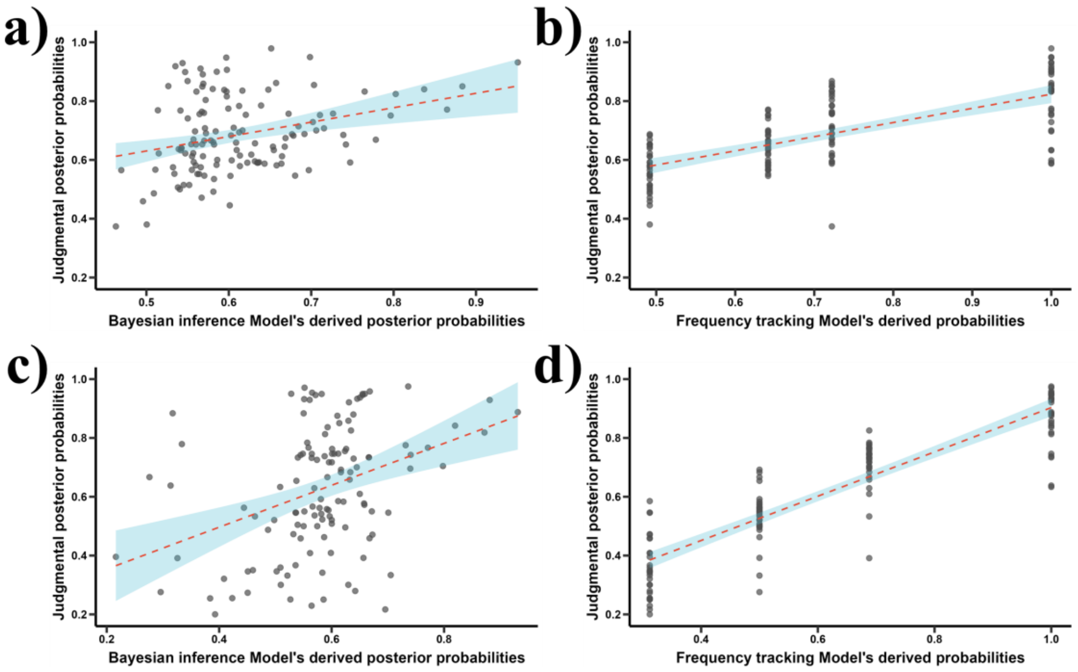

Supplementary Figure 2. Scatter plots of predicted probabilities versus participants' actual judgments.

Note. a) and c) show the predicted probabilities from the Bayesian inference model for Experiment 1 and Experiment 2, respectively. b) and d) show the predicted probabilities from the Frequency tracking model for Experiment 1 and Experiment 2, respectively. In each panel, the dashed line represents the fitted regression line, and the shaded area indicates the 95% confidence interval.

To demonstrate that the Bayesian inference model can better capture the variability in participants' responses under the same condition, the experimental

condition was also included as a random variable in the Bayesian hierarchical linear regression. The results showed that the Frequency tracking model could no longer positively predict participants' responses (Experiment 1: $\beta = 0.40$, 95% CrI = [-3.31, 4.80], *Pr* ($\beta > 0$) = 78.20%, $\delta_t = 2.94$; Experiment 2: $\beta = 0.87$, 95% CrI = [-2.36, 3.91], *Pr* ($\beta > 0$) = 86.50%, $\delta_t = 3.08$), whereas the Bayesian inference model continued to do so (Experiment 1: $\beta = 0.49$, 95% CrI = [-0.04, 0.99], *Pr* ($\beta > 0$) = 96.80%, $\delta_t = 1.64$; Experiment 2: $\beta = 0.31$, 95% CrI = [-0.02, 0.63], *Pr* ($\beta > 0$) = 96.90%, $\delta_t = 0.86$).

In short, the Bayesian inference model provided a better prediction of participants' actual responses compared to the Frequency tracking model. This offers evidence that participants' inference of group norms is a Bayesian process that integrates both prior beliefs and observed evidence.

4 Analysis controlling for gender and age

To examine whether participants' gender and age influenced the observed effects, data on prior probability, posterior probability, and Kullback–Leibler (KL) divergence from all three experiments were analyzed using the afex package (Singmann et al., 2017), with age included as a covariate and gender treated as a between-subjects factor.

4.1 Experiment 1

Prior Probabilities. When including gender as a between-subjects factor and age as a covariate, the main effect of the number of norm-consistent behaviors on

prior probability was not significant, F (2.10, 48.31) = 0.73, $p = 0.491$. Neither gender (F (1, 23) = 1.41, $p = 0.246$) nor age (F (7, 23) = 1.79, $p = 0.137$), nor their interactions with the number of norm-consistent behaviors (gender × the number of norm-consistent behaviors: F (2.10, 48.31) = 0.10, $p = 0.916$; age × the number of norm-consistent behaviors: F (14.70, 48.31) = 0.72, $p = 0.750$) reached significance. These findings indicate that the effect of the number of norm-consistent behaviors on prior probability was not influenced by participants' gender or age.

Posterior Probabilities. When including gender as a between-subjects factor and age as a covariate, the main effect of the number of norm-consistent behaviors on posterior probability was highly significant, F (1.48, 34.01) = 32.58, $p < 0.001$, $\eta_p^2 = 0.59$. Neither gender (F (1, 23) = 0.06, $p = 0.815$) nor age (F (7, 23) = 0.54, $p = 0.793$), nor their interactions with the number of norm-consistent behaviors (gender × the number of norm-consistent behaviors: F (1.48, 34.01) = 0.44, $p = 0.590$; age × the number of norm-consistent behaviors: F (10.35, 34.01) = 0.97, $p = 0.491$) reached significance. These findings indicate that the observed effect of the number of norm-consistent behaviors on posterior probability was robust and did not differ as a function of participants' gender or age.

Updating degrees of norm-related belief. When including gender as a between-subjects factor and age as a covariate, the main effect of the number of norm-consistent behaviors on updating degrees of norm-related belief (i.e., D_{KL}) was significant, F (1.22, 27.97) = 24.22, $p < 0.001$, $\eta_p^2 = 0.51$. Neither gender (F (1, 23) = 0.58, $p = 0.456$) nor age (F (7, 23) = 0.76, $p = 0.623$), nor their interactions with the

number of norm-consistent behaviors (gender × the number of norm-consistent behaviors: F (1.22, 27.97) = 0.22, p = 0.693; age × the number of norm-consistent behaviors: F (8.51, 27.97) = 1.08, p = 0.409) reached significance. These findings indicate that the observed effect of the number of norm-consistent behaviors on D_{KL} was robust and did not differ as a function of participants' gender or age.

4.2 Experiment 2

Prior Probabilities. When including gender as a between-subjects factor and age as a covariate, the main effect of the proportion of norm-consistent behaviors on prior probability was not significant, F (1.57, 31.34) = 0.05, p = 0.913. Neither gender (F (1, 20) = 2.11, p = 0.162) nor age (F (8, 20) = 0.38, p = 0.918), nor their interactions with the proportion of norm-consistent behaviors (gender × the proportion of norm-consistent behaviors: F (1.57, 31.34) = 2.86, p = 0.084; age × the proportion of norm-consistent behaviors: F (12.53, 31.34) = 1.02, p = 0.457) reached significance. These findings indicate that the effect of the proportion of norm-consistent behaviors on prior probability was not influenced by participants' gender or age.

Posterior Probabilities. When including gender as a between-subjects factor and age as a covariate, the main effect of the proportion of norm-consistent behaviors on posterior probability was highly significant, F (1.45, 29.02) = 237.92, $p < 0.001$, $\eta_p^2 = 0.92$. Neither gender (F (1, 20) = 0.50, p = 0.488) nor age (F (8, 20) = 0.84, p = 0.581), nor their interactions with the proportion of norm-consistent behaviors (gender × the proportion of norm-consistent behaviors: F (1.45, 29.02) = 1.72, p = 0.202; age

× the proportion of norm-consistent behaviors: F (11.61, 29.02) = 1.53, p = 0.170) reached significance. These findings indicate that the observed effect of the proportion of norm-consistent behaviors on posterior probability was robust and did not differ as a function of participants' gender or age.

Updating degrees of norm-related belief. When including gender as a between-subjects factor and age as a covariate, the main effect of the proportion of norm-consistent behaviors on updating degrees of norm-related belief (i.e., D_{KL}) was significant, F (1.63, 32.59) = 55.61, $p < 0.001$, $\eta_p^2 = 0.74$. Neither gender (F (1, 20) = 0.18, p = 0. 673) nor age (F (8, 20) = 0.50, p = 0.844), nor their interactions with the proportion of norm-consistent behaviors (gender × the proportion of norm-consistent behaviors: F (1.63, 32.59) = 0.49, p = 0.578; age × the proportion of norm-consistent behaviors: F (13.03, 32.59) = 0.82, p = 0.636) reached significance. These findings indicate that the observed effect of the proportion of norm-consistent behaviors on D_{KL} was robust and did not differ as a function of participants' gender or age.

4.3 Experiment 3

Prior Probabilities. When including gender as a between-subjects factor and age as a covariate, the main effect of the proportion of norm-consistent behaviors on prior probability was not significant, F (2.30, 50.69) = 0.22, p = 0.832. Neither gender (F (1, 22) = 0.23, p = 0.639) nor age (F (8, 22) = 0.36, p = 0.929), nor their interactions with the proportion of norm-consistent behaviors (gender × the proportion of norm-consistent behaviors: F (2.30, 50.69) = 0.34, p = 0.741; age × the proportion of norm-consistent behaviors: F (18.43, 50.69) = 0.56, p = 0.915) reached

significance. These findings indicate that the effect of the proportion of norm-consistent behaviors on prior probability was not influenced by participants' gender or age.

Posterior Probabilities. When including gender as a between-subjects factor and age as a covariate, the main effect of the proportion of norm-consistent behaviors on posterior probability was highly significant, F (1.60, 35.23) = 149.04, $p < 0.001$, $\eta_p^2 = 0.87$. Neither gender (F (1, 22) = 0.00, $p = 0.981$) nor age (F (8, 22) = 1.54, p = 0.199), nor their interactions with the proportion of norm-consistent behaviors (gender × the proportion of norm-consistent behaviors: F (1.60, 35.23) = 1.48, $p = 0.241$; age × the proportion of norm-consistent behaviors: F (12.81, 35.23) = 1.05, $p = 0.426$) reached significance. These findings indicate that the observed effect of the proportion of norm-consistent behaviors on posterior probability was robust and did not differ as a function of participants' gender or age.

Updating degrees of norm-related belief. When including gender as a between-subjects factor and age as a covariate, the main effect of the proportion of norm-consistent behaviors on updating degrees of norm-related belief (i.e., D_{KL}) was significant, F (1.63, 35.76) = 22.35, $p < 0.001$, $\eta_p^2 = 0.50$. Neither gender (F (1, 22) = 1.40, p = 0. 250) nor age (F (8, 22) = 0.51, $p = 0.838$), nor their interactions with the proportion of norm-consistent behaviors (gender × the proportion of norm-consistent behaviors: F (1.63, 35.76) = 0.19, $p = 0.782$; age × the proportion of norm-consistent behaviors: F (13.03, 35.76) = 0.61, $p = 0.832$) reached significance. These findings indicate that the observed effect of the proportion of norm-consistent behaviors on

D_{KL} was robust and did not differ as a function of participants' gender or age.

5 Computational details for three Bayesian network models

The three network models (IE, FC, and DM) fundamentally differ in their assumptions about the causal relationships between norms (*N*), desires (*D*), and actions (*A*). These distinct theoretical assumptions are formally represented by their respective joint probability distributions, which form the core statistical basis for each model. The specific joint probability distributions are as follows:

Independent Effect (IE) Model: This model assumes that norms (*N*) and desires (*D*) are independent but jointly influence actions (*A*).

$P(N, D, A) = P(N) \cdot P(D) \cdot \mathrm{P}(A \mid D, N)$

Fully Connected (FC) Model: This model hypothesizes that norms (N) causally influence desires (D), and both norms and desires jointly determine actions (A).

$P(N, D, A) = P(N) \cdot P(D \mid N) \cdot \mathrm{P}(A \mid D, N)$

Desire Mediation (DM) Model: This model posits that the influence of norms (N) on actions (A) is fully mediated by desires (D).

$P(N, D, A) = P(\mathrm{N}) \cdot P(D \mid N) \cdot P(A \mid D)$

6 Desire-related prior and posterior probabilities

6.1 Desire-related prior probabilities

To demonstrate the differences between measures of group norms and desires, desire-related prior probabilities were also compared across different conditions. The prior probabilities under each condition are shown in Supplementary Figure 3. One-

way repeated measures ANOVA indicated that the main effect of the proportion of norm-consistent behaviors was not significant, F (2.51, 77.83) = 0.64, p = 0.562. Additionally, one-sample t-tests were conducted for comparing the prior probabilities under each condition to the random level (0.5). The results showed that there were no significant differences between the prior probabilities and the random level in any condition, ts < 2.99, ps > 0.065.

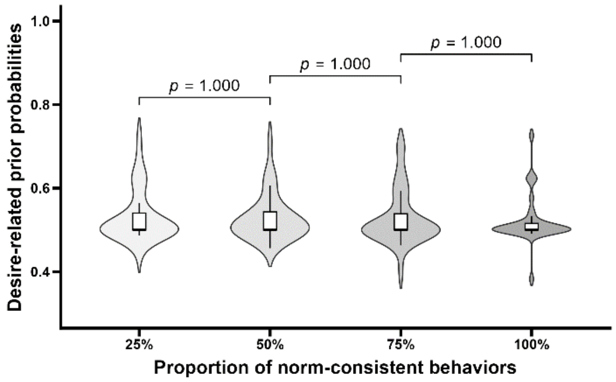

Supplementary Figure 3. Desire-related prior probabilities as a function of the proportion of norm-consistent behaviors

6.2 Desire-related posterior probabilities

Similarly, desire-related posterior probabilities were also compared across all conditions, as shown in Supplementary Figure 4. One-way repeated measures ANOVA indicated that the main effect of the proportion of norm-consistent behaviors was significant, F (1.23, 38.03) = 10.05, p = 0.002, η_p^2 = 0.25. Further post-hoc comparisons revealed that the desire-related posterior probability at 100% norm-consistent behaviors was significantly greater that at 75%, t (31) = 3.06, p = 0.027, Cohen's d = 0.27. There were no significant differences in the desire-related posterior

probabilities between other adjacent conditions, *t*s < 2.69, *p*s > 0.069。

At the same time, one-sample *t*-tests were conducted for comparing the desire-related posterior probabilities under each condition to the random level (0.5). The results showed that the desire-related posterior probabilities under 25% norm-consistent behaviors did not differ significantly from the random level, *t* (31) = 1.30, *p* = 1.000 (corrected *p* values). In contrast, the desire-related posterior probabilities under the other conditions were significantly greater than the random level, *t*s > 6.41, *p*s < 0.001, Cohen's *d*s > 1.13.

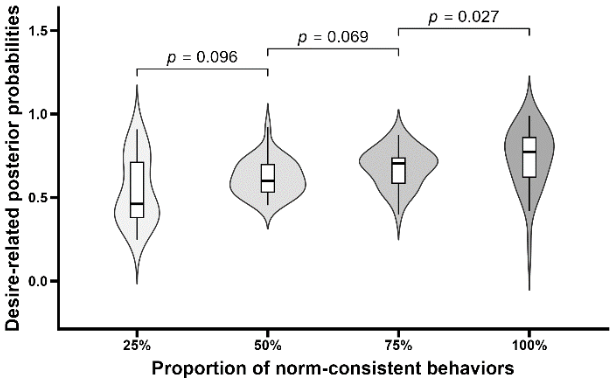

Supplementary Figure 4. Desire-related posterior probabilities as a function of the proportion of norm-consistent behaviors

References

Albarracín, D., Sunderrajan, A., Dai, W., & White, B. X. (2019). The social creation of action and inaction: From concepts to goals to behaviors. In *Advances in experimental social psychology* (Vol. 60, pp. 223–271). Academic Press.

Baker, A., Sharma, K., Dunham, Y., & Jara-Ettinger, J. (2025). People use mixed

strategies to make efficient but structured inferences about agents in roles. In *Proceedings of the Annual Meeting of the Cognitive Science Society* (Vol. 47).

Singmann, H., Bolker, B., Westfall, J., & Aust, F. (2017). afex: Analysis of factorial experiments. (R package version 0.18-0. http://cran.r-project.org/package=afex)
